# Using Expert Elicitation to Adjust Published Intervention Effects to Reflect the Local Context

**DOI:** 10.1177/23814683231226335

**Published:** 2024-01-25

**Authors:** Jodi Gray, Tilenka R. Thynne, Vaughn Eaton, Rebecca Larcombe, Mahsa Tantiongco, Jonathan Karnon

**Affiliations:** Flinders Health and Medical Research Institute (FHMRI), College of Medicine and Public Health, Flinders University, Bedford Park, SA, Australia; Flinders Medical Centre, Southern Adelaide Local Health Network (SALHN), Bedford Park, SA, Australia; College of Medicine and Public Health, Flinders University, Bedford Park, South Australia, Australia; SA Pharmacy Southern Adelaide Local Health Network (SALHN), Department of Health and Wellbeing, SA Health, Government of South Australia, Bedford Park, SA, Australia; SA Pharmacy Southern Adelaide Local Health Network (SALHN), Department of Health and Wellbeing, SA Health, Government of South Australia, Bedford Park, SA, Australia; SA Pharmacy Southern Adelaide Local Health Network (SALHN), Department of Health and Wellbeing, SA Health, Government of South Australia, Bedford Park, SA, Australia; Flinders Health and Medical Research Institute (FHMRI), College of Medicine and Public Health, Flinders University, Bedford Park, SA, Australia

**Keywords:** structured expert elicitation, local health service evaluation, health services research, hypoglycemia, hospital-acquired complication

## Abstract

**Background.** Local health services make limited use of economic evaluation to inform decisions to fund new health service interventions. One barrier is the relevance of published intervention effects to the local setting, given these effects can strongly reflect the original evaluation context. Expert elicitation methods provide a structured approach to explicitly and transparently adjust published effect estimates, which can then be used in local-level economic evaluations to increase their local relevance. Expert elicitation was used to adjust published effect estimates for 2 interventions targeting the prevention of inpatient hypoglycemia. **Methods.** Elicitation was undertaken with 6 clinical experts. They were systematically presented with information regarding potential differences in patient characteristics and quality of care between the published study and local contexts, and regarding the design and application of the published study. The experts then assessed the intervention effects and provided estimates of the most realistic, most pessimistic, and most optimistic intervention effect sizes in the local context. **Results.** The experts estimated both interventions would be less effective in the local setting compared with the published effect estimates. For one intervention, the experts expected the lower complexity of admitted patients in the local setting would reduce the intervention’s effectiveness. For the other intervention, the reduced effect was largely driven by differences in the scope of implementation (hospital-wide in the local setting compared with targeted implementation in the evaluation). **Conclusions.** The pragmatic elicitation methods reported in this article provide a feasible and acceptable approach to assess and adjust published intervention effects to better reflect expected effects in the local context. Further development and application of these methods is proposed to facilitate the use of local-level economic evaluation.

**Highlights:**

- Local health services make limited use of economic evaluation to inform their decisions on the funding of new health service interventions. One barrier to use is the relevance of published intervention evaluations to the local setting.
- Expert elicitation methods provide a structured way to consider differences between the evaluation and local settings and to explicitly and transparently adjust published effect estimates for use in local economic evaluations.
- The pragmatic elicitation methods reported in this article offer a feasible and acceptable approach to adjusting published intervention effects to better reflect the effects expected in the local context. This increases the relevance of economic evaluations for local decision makers.

## Introduction

Local health services are defined as organizations with an allocated budget for the provision of health services within a defined geographical area. Every local health service is different, with respect to current and expected demand (reflecting population needs and preferences) and supply (reflecting availability and costs of resources). There is a large but underutilized evidence base on the effectiveness of health service interventions.^1^ This could be better used to inform health service planning, which is an ongoing process for local health services. However, a key issue is the interpretation of published evidence from a local health service perspective. In addition to potential biases associated with study design and application, there is also the question of whether an intervention is expected to have the same effects in the local context compared with the context in which it was evaluated.

Expert elicitation methods provide a structured approach to the explicit and transparent assessment of evidence to estimate uncertain or unobserved parameters.^2,3^ Elicitation has predominantly been used in the context of decision analytic modeling for the economic evaluation of health technologies, mostly to estimate extrapolation parameters (e.g., probabilities of adverse events) but not to estimate intervention effect parameters. An example of elicitation used to estimate intervention effects is found in a preimplementation economic evaluation of a service delivery intervention to improve clinical handovers.^4^ After designing the intervention, Yao et al.^4^ elicited expected effects from experts, but this was done without reference to existing evidence on the effects of similar interventions.

Bojke et al.^2,3^ provided a reference protocol for the use of expert elicitation to inform health care decision making, which incorporates flexibility with respect to the choice of methods and their adaptation for use in different contexts. Bojke et al. advocated for the use of elicitation in economic evaluations to inform local decision making (e.g., in evaluations undertaken for Clinical Commissioning Groups in the UK’s National Health Service [NHS]). However, in an extensive review undertaken during the protocol’s development, they found no examples of elicitation used in the context of local-level evaluations.

This article describes the use of expert elicitation methods to estimate the expected effects of intervention options. It was undertaken as one step in a local-level economic evaluation of interventions to prevent hypoglycemia as a hospital-acquired complication. The complete local-level economic evaluation framework and the evaluation findings are described separately.^5^

## Methods

### The Broader Project: Local-Level Economic Evaluation Overview

The Southern Adelaide Local Health Network (SALHN) is a local health service in the Adelaide metropolitan area. It manages 2 acute care public hospitals: Flinders Medical Centre (FMC) and Noarlunga Hospital. In 2019, SALHN identified that reducing hospital-acquired hypoglycaemic events at FMC was a priority. A working group was established to undertake a local-level economic evaluation of health service delivery models to inform local decision making on this priority. The evaluation used a 10-step framework:

1. Define the objective of the local-level economic evaluation
2. Form an evaluation working group and define the Population, Intervention, Comparator, and Outcomes (PICO)
3. Analyze local data to describe the current context (e.g., identify root causes and determine baseline event rates)
4. Conduct a pragmatic literature review to identify intervention options
5. Perform a working group assessment (and shortlisting) of the intervention options
6. Conduct a preliminary modeled local-level economic evaluation (cost-consequence analysis)
7. Working group rule-out intervention options
8. Elicit expected intervention effect(s) in the local context
9. Final modeled local-level economic evaluation (cost-consequence analysis)
10. Working group to make recommendations to local decision makers

The working group included clinical staff, including 3 pharmacists (V.E., R.L., M.T.), a credentialed diabetes nurse educator, and an endocrinologist (T.T.), who worked together managing diabetes across SALHN, as well as members of the SALHN quality improvement team (with a nursing background) and data analytics team, and 2 academic health economists (J.G., J.K.). The clinical and quality improvement SALHN staff were the experts from whom relevant parameter values were elicited, as described in this article.

Health service delivery models are used to organize the effective delivery of health care. Based on a pragmatic literature review of potential delivery model intervention options^6^ and a preliminary assessment of expected costs and benefits, the working group selected 2 interventions to be included in the final local-level economic analysis:

1. Virtual glycemic management service (vGMS): A daily report of all inpatients with dysglycemia in the last 24 h was generated. Endocrinologists, certified diabetes educator pharmacists, and certified diabetes educator nurses in the vGMS team remotely reviewed the reported patients’ electronic medical records (EMRs) and added recommendations on glycemic management before morning rounds.^7,8^
2. Root-cause survey: Nurses completed an automated pop-up survey to identify the cause(s) of hypoglycemic events soon after the events occurred. The survey acted as a reflective intervention as well as identifying root causes for brief, targeted educational presentations.^9^

Expert elicitation was undertaken to adjust the published effect estimates to reflect the expected effects of the interventions in the local setting. These elicited estimates were then used in the final local-level economic evaluation.^5^ The current article reports on the expert elicitation process.

### Elicited Quantities

The vGMS study reported event rates as the number of hypoglycemic events per 100 patient-days, for example, pre- and postimplementation rates of 0.78 and 0.49 patient days with a hypoglycemic event per 100 patient-days, respectively.^7,8^ The root-cause survey study reported event rates as the number of hypoglycemic test results per 100 point-of-care blood glucose level (PoC-BGL) tests.^9^ There were differences in the preimplementation (baseline) rates between the study settings and the local settings, which made the direct elicitation of an expected postimplementation rate too complicated (i.e., the experts would have to account for baseline rate differences in defining their expected postimplementation values).

Relative risks (RRs) for each intervention had been used in the preliminary assessment of expected costs and benefits. The final local-level economic evaluation would also use RRs for each intervention.^5^ The RRs would be applied to a patient-level data set that reflected the local setting’s baseline hypoglycemia event rates. This would enable the number of hypoglycemic events (and the number of patients experiencing an event) to be estimated for each intervention. RRs were therefore the most fit-for-purpose quantities to elicit. In addition, this measure could be presented to the experts in a format (both text-based and graphical) that could be intuitively understood and conceptualized.

**Figure 1:**
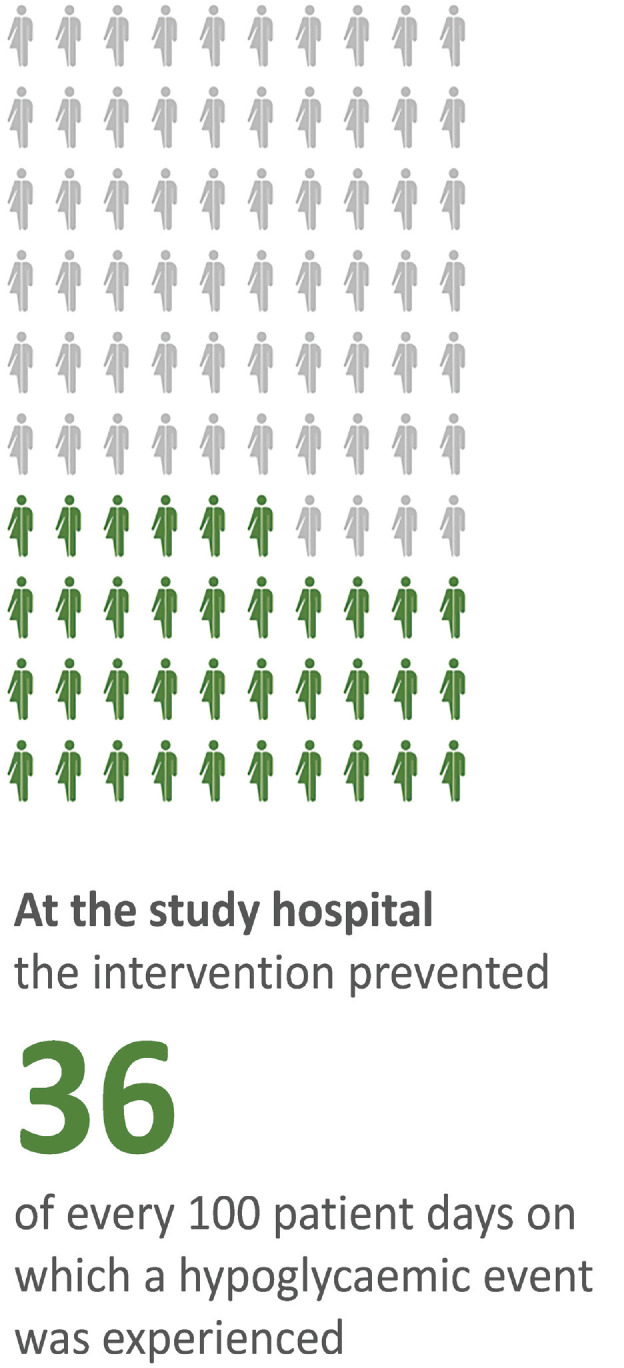
Format used to present the intervention effects to the experts.

The published RRs were used to indicate the number of baseline hypoglycemic events (or baseline hypoglycemic patient-days) that would be prevented following implementation of the intervention. Figure 1 shows how this was presented to the experts for the vGMS intervention, where the RR of a patient-day with a hypoglycemic event was 0.64 (i.e., 36 out of every 100 patient-days with an event were avoided at the study hospital).^7,8^ For the root-cause intervention, a similar figure was used to show that 32 out of every 100 hypoglycemic events (i.e., PoC-BGL test measurements) at baseline were prevented following intervention implementation at the study hospital.^9^ Both interventions reported unadjusted RRs for hypoglycemia.^7–9^ The vGMS study reported a separate unadjusted RR for severe hypoglycaemia.^7,8^

### Expert Elicitation Overview

The elicitation process was guided by the reference protocol developed by Bojke et al.^2,3^

Elicitation was undertaken with the members of the working group only. Input from additional experts was considered unnecessary given the established nature of the working group, the relevant and diverse clinical expertise within the group, the strong working relationships between the group members, and the time constraints of experts (due to COVID-19). A PowerPoint presentation was developed by the 2 academic health economists (J.G., J.K.) to present the assembled evidence and to guide the experts through the elicitation process (see Supplementary Materials A for the full presentation). The presentation went through multiple iterations and was piloted with peer researchers before it was deemed ready to use.

The elicitation process was facilitated by the 2 academic health economists (J.G., J.K.). It guided the experts to consider the potential effects of 3 broad contextual factors on the expected intervention effects in the local context: patient complexity, quality of care, and potential biases associated with the research study design and application. Evidence to inform expert judgments regarding differences in patient complexity between the published study and the local patient populations was assembled to assess the comparability of the Population element of the PICO (Population Intervention Comparator Outcome) statement. Quality of care at the study sites in the period prior to the implementation of the evaluated interventions was compared with the current quality of care at the local site to assess the comparability of the Comparator element of the PICO statement. Potential biases associated with the research study design and application were assessed, specifically regarding the observational study designs and differences in the scale and scope of implementation between the published study and the local contexts.

Information collected on the 3 contexts is summarized in Tables 1 and 2 (and in Supplementary Materials A). Information on the local context was compiled from a clinical audit of inpatients who experienced hypoglycemia, data extracted from local health system databases, and general information on the local health service. For the published study sites, relevant information was extracted from the study hospital’s Web site and the published evaluation, including baseline infrastructure and protocols relating to the management of hypoglycemia, baseline events rates, and study design.

**Table 1:** Contextual Information for Intervention Option 1: Virtual Glycemic Management System (vGMS)

	Evaluation Context	Local Context (FMC)
Hospital context		
Location	San Francisco, California, USA	Adelaide, South Australia, Australia
Data years	2012–2015	2019
Hospital type	Quaternary care, academic and VA	Principal referral hospital
Infrastructure	Diabetes committee that provided oversight	
	EMR was newly implemented in the months before baseline	EMR recently implemented^a^
	Computerized insulin orders sets were available for eating, NPO, and IV insulin	Hospital protocols guide use of basal bolus insulin in hospitalized patients^a^; insulin order sets available^a^
	Automatic, real-time upload of PoC-BGLs into the EMR	PoC-BGLs manually entered into EMR^a^
PoC-BGL testing frequency	5 per day if eating	4 to 5 per day if eating or fasting^a^
	6 per day if NPO or on total parenteral or enteral nutrition	
	Hourly if on IV insulin infusion	Hourly if on IV insulin infusion^a^
Personnel	Experienced staff (endocrinologists, CDE nurses, CDE pharmacists) managed inpatient diabetes	Experienced team of endocrinologists and CDE nurses provide a consult service for admitted patients with diabetes within SALHN^a^
	Medical, nursing, and pharmacy staff were well-trained in insulin pharmacodynamics and inpatient insulin regimens and were receptive to recommendations	
Ranking	UCSF Health is ranked the #1 hospital in California, #6 in the United States, and in the top 10 US hospitals for diabetes and endocrinology	
Patient context		
General information	Quaternary care is the highest designation for facilities that treat the most complex and specialized conditions	
	UCSF provides more hospital care for Medicaid patients than any other hospital in San Francisco	
Baseline rates^b^		
% with a PoC-BGL measured	29	26
Mean LOS (d)	8.4	4.3
Hypoglycemia rate (per 100 patient-days)	0.78	0.53
Severe hypoglycemia rate (per 100 patient-days)	0.032	0.054
Research context^c^		
Study design	Observational design included all eligible adult patients admitted during the study time period; outcomes were evaluated using routinely collected EMR data	
Buy-in	Intervention was designed and implemented by clinicians at the hospital and therefore may have strong buy-in from the vGMS team, endocrinology, management, and hospital staff	Intervention would not be implemented without strong buy-in from the endocrinology team^a^

CDE, certified diabetes educator; EMR, electronic medical record; FMC, Flinders Medical Centre; IV, intravenous; LOS, length of stay; NPO, nil by mouth; PoC-BGL, point-of-care blood glucose level; SALHN, Southern Adelaide Local Health Network; UCSF, University of California San Francisco; VA, Veterans Affairs.

a Indicates additional information provided by the working group during elicitation discussions.

b EMRs were not initially in use at the main local hospital (FMC). Therefore, baseline event rates for FMC were extrapolated using EMR PoC-BGL data from the smaller local hospital (Noarlunga Hospital).

c Potential biases associated with the research study design and application.

**Table 2:** Contextual Information for Intervention Option 2: Root-Cause Survey with Targeted Education

	Evaluation Context	Local Context (FMC)
Hospital context		
Location	New York (City), New York, USA	Adelaide, South Australia, Australia
Data years	2016–2017	2019
Hospital type	Academic medical center	Principal referral hospital
Infrastructure	EMR (Sunrise)	EMR (Sunrise) recently implemented^a^
	Real-time automatic upload of PoC-BGLs into the EMR	PoC-BGLs manually entered into the EMR^a^
	Glycemic control dashboard in the EMR showing BGLs and insulin dose in 4 time buckets (meals, overnight)	
Ranking	New York-Presbyterian (Weill Cornell Medical Center) is ranked the #1 hospital in New York, #7 hospital in the United States, and #5 in the United States for diabetes and endocrinology	
	National recognition for excellence in nursing (in top 8% of US hospitals)	
Patient context		
Intervention implemented in	2 general medical wards only	Hospital-wide
Baseline rates^b^		
Hypoglycemia rate (per 100 BGL measurements)	2.27	1.17
Research context^c^		
Study design	Observational design included all eligible adult patients admitted to the study wards during the study time period; outcomes were evaluated using routinely collected EMR data	
Buy-in	Intervention was designed and implemented by clinicians at the hospital and therefore may have strong buy-in from management and hospital staff	

EMR, electronic medical record; FMC, Flinders Medical Centre; PoC-BGL, point-of-care blood glucose level.

a Indicates additional information provided by the working group during elicitation discussions.

b EMRs were not initially in use at the main local hospital (FMC). Therefore, baseline event rates for FMC were extrapolated using EMR PoC-BGL data from the smaller local hospital (Noarlunga Hospital).

c Potential biases associated with the research study design and application.

Two elicitation sessions were held. The first session was both in-person and online, while the second session was online only. The aim was to elicit local treatment effect parameters in 2 sessions, with 1 session for each intervention option. However, areas for improvement were identified during the first session, which informed modifications to the elicitation process. In the second session, the revised format was applied to both intervention options.

### Original Elicitation Process

The experts were sequentially presented with the assembled comparative information on each of these 3 contextual factors (patient complexity, quality of care, and potential biases associated with the research study design and application, as summarized in Tables 1 and 2). They were asked to reflect on if and how each factor affected the expected effectiveness of the intervention in the local context. The group were asked to answer the following questions relating to the 3 contextual factors, respectively:

Patient complexity:

- “Are the study hospital patients more, less or the same complexity as local patients?”
- “Thinking about the complexity of patients, do you think the intervention would prevent more, less or the same number of patient days with hypoglycaemia per 100 patient days with hypoglycaemia as reported in the published evaluation?”

Hospital setting (quality of care):

- “Is quality of care at the study hospital more, less or the same as at the local hospital?”
- “Thinking about quality of care, do you think the intervention would prevent more, less or the same number of patient days with hypoglycaemia per 100 patient days with hypoglycaemia as reported in the published evaluation?”

Potential biases associated with the research study design and application (i.e., “research context”):

- “Thinking about the research context, do you think the intervention would prevent more, less or the same number of patient days with hypoglycaemia per 100 patient days with hypoglycaemia as reported in the published evaluation?”

The group were then provided with a summary of their responses to the above questions, and based on the variable interval method,^2,3^ the experts were asked to provide a range of plausible values for the intervention’s effect in the local setting, before providing a best estimate. Specifically, the group were shown Figure 1, representing the intervention effects in the study context, and were asked, “Thinking about the complexity of patients and the quality of care and the research context at the local hospital compared to the study hospital, at most, how many out of every 100 patient days on which a hypoglycaemic event was experienced would be prevented in the local hospital?” followed by the same question but asking for the groups’“at least” and “best” estimates.

### Revised Elicitation Process

During session 1, it became apparent that the comparison of the intervention study’s baseline hypoglycemic event rates and local event rates was central to the experts’ comparisons of hospital setting (quality of care) and patient complexity; that is, differences in baseline event rates were viewed as being representative of potential variation in patient complexity and quality of care. As a result, a revised elicitation approach was developed and applied in the second elicitation session.

The revised elicitation approach discussed potential drivers of differences in baseline hypoglycemic event rates (patient complexity and quality of care) and the effects of potential biases associated with the research study design and application as separate issues, prior to asking questions based on the variable interval method to generate a range of plausible values for the interventions’ effects in the local setting.

The expert group were presented with the assembled comparative information on the hospital settings and patient characteristics (as summarized in Tables 1 and 2), followed by a comparison of baseline hypoglycemic events. Potential scenarios relating to the rationale for the reported differences in hypoglycemia event rates were presented to the experts, to promote discussion around whether such differences imply the intervention would be more or less effective in the local context.

The scenarios included the following:

Scenario A:

- At baseline, the local site is better at preventing hypoglycemia events in lower complexity patients, so more complex patients experienced hypoglycemic events at the local site compared with the study hospital.
- The intervention is expected to prevent a lower proportion of events in the more complex patients.

Scenario B:

- At baseline, the local hospital treated less complex patients than the study site did.
- The intervention is expected to prevent a higher proportion of events in less complex patients.

Scenario C (root-cause survey only):

- The evaluated intervention was implemented only in general medical wards, but the intervention will be implemented hospital-wide in the local setting.
- Is the intervention is expected to prevent more, less, or the same proportion of events if implemented hospital-wide?

After discussing the scenarios, the experts were asked to respond to the following statement: “Thinking about the complexity of patients and the quality of care in the local setting compared to the study hospital, do you think the intervention would prevent more, less or the same number of hypoglycaemic events per 100 hypoglycaemic events in the local setting as reported in the published evaluation?”

Information relating to potential biases associated with the research study design and application was then presented and discussed by the experts. They were then asked to respond to the following statement: “Thinking about the research context at the study hospital, do you think the intervention would prevent more, less or the same number of hypoglycaemic events per 100 hypoglycaemic events in the local setting as reported in the published evaluation?”

Following this, the experts were asked to provide consensus-based values for the most optimistic, most pessimistic, and most realistic estimate of how many out of 100 hypoglycemic events would be prevented if the intervention were to be implemented in the local context.

### Ethical Statement

The SALHN Office for Research deemed the hospital-acquired hypoglycemia prevention project to be a quality improvement activity; therefore, ethical approval was not required.

## Results

Four members of the working group attended the first elicitation session (3 in-person, 1 online via Microsoft Teams). They were 2 pharmacists (V.E., R.L.), an endocrinologist (T.T.), and a member of the SALHN quality improvement team who had a nursing background. All experts were based within SALHN and worked within the hospital setting. In this session, the vGMS intervention effect estimates were elicited using the original elicitation process.

Six members attended the second session (all online via Microsoft Teams). They were 3 pharmacists (V.E., R.L., M.T.), a credentialled diabetes nurse educator, an endocrinologist (T.T.), and a member of the SALHN quality improvement team who had a nursing background. All experts were based within SALHN and worked within the hospital setting. In this session, local effect estimates for the vGMS intervention were reviewed and estimates of the root-cause survey with targeted education intervention were elicited using the revised elicitation process.

### Intervention Option 1: vGMS Intervention

In the first elicitation session, the following observations were made by the experts with respect differences in patient complexity, quality of care, and potential biases associated with the research study design and application:

Patient complexity:

- The experts wondered if differences in funding models between the United States and Australia would influence how sick patients became before reaching the hospital. While they considered whether there would be differences in health care access (given Australia has a universal, publicly funded health care system and the United States does not), the experts did not reach a conclusion on this.
- The experts initially thought the quaternary care designation (US terminology) of the study hospital would indicate a similar level of patient complexity to their Australian tertiary care hospital (the local hospital). However, the presented rankings of the evaluation hospital (e.g., number 1 in California) and the larger catchment area (Northern California: 15.4 million; SALHN: 376,140) suggested that the study hospital was likely to receive more referrals for more complex patients from a broad geographical area.
- The data used to estimate local hypoglycemic event rates may have underestimated the true event rates and hence underrepresented the complexity of local patients.

Quality of care:

- Protocol-related information was considered with respect to quality of care. For example, the experts noted that international recommendations on target PoC-BGL ranges had been revised since the time of the vGMS evaluation to reduce the risk of hypoglycemia (and these revisions had been implemented locally). In 2012 to 2015, at the time of the evaluation, the recommended target was generally <7.8 mmol/L (140 mg/dL) for most hospitalized patients.^10,11^ However, by 2019, greater recognition of the risk of hypoglycemia led to a revised target of generally 7.8 to 10 mmol/L (140–180 mg/dL).^12^ This change may partially explain the lower baseline event rates in the local context.
- Differences in recommendations regarding the frequency of PoC-BGL testing were discussed, noting that test frequency may be slightly lower in the local context for those not on IV insulin (4–5 tests per day locally compared with 5–6 per day in the study setting).

Potential biases associated with the research study design and application:

- The observational study design did not present a significant risk of selection or information bias primarily because the evaluation used routinely collected data and the cohort included all eligible patients admitted to the hospital during the relevant time periods (researcher’s observation).
- The vGMS intervention would require significant sustained resources and support from the endocrinology team and therefore would not be implemented without strong buy-in from that team. This led to the conclusion that a similar level of buy-in was expected in the study and local context.

The experts concluded that there was no expectation of differences in quality of care between the evaluation and local contexts. However, the experts thought that patient complexity may be slightly higher at the study hospital, primarily based on the status of the study hospital (number 1 in California) and the expectation that they received referrals of complex patients from a large geographical area. Lower baseline hypoglycemic event rates in the local setting were attributed to a lower patient complexity in the local setting. Potential biases associated with the research study design and application were not expected to influence the published intervention effect estimates in the local context. From this, the experts concluded that the intervention would be less effective in the local setting. They estimated the intervention effect on hypoglycemia in the local setting to be a RR of 0.76 (range: 0.70–0.82) compared with the published RR of 0.64 (Table 3).

**Table 3:** Published and Elicited Effect Estimates (Relative Risks) for the Interventions of Interest^a^

	Hypoglycemia	Severe Hypoglycemia
vGMS		
Published estimate of RR^10,11^	0.64 (95% CI: 0.57 to 0.70)	0.31 (95% CI: 0.15 to 0.59)
Elicited estimate of RR	0.76 (range: 0.70 to 0.82)	0.50 (range: 0.20 to 0.75)
Root-cause survey with targeted education		
Published estimate of RR^12^	0.68 (95% CI: NR)	NR
Elicited estimate of RR	0.85 (range: 0.80 to 0.90)	0.85 (range: 0.80 to 0.90)

CI, confidence interval; NR, not reported; RR, relative risk; vGMS, virtual glycemic management service.

a The 95% CIs were not presented for published RRs during the elicitation. Elicited estimates are the most realistic estimates, with ranges defined by the most optimistic to most pessimistic estimates.

For the severe-hypoglycaemic event outcome, a higher baseline event rate in the local setting led the working group to theorize that either:

- There is greater scope for improvement in the local setting and the intervention effect could be larger than the published estimate, or
- The local setting has a lower baseline quality of care and the intervention effect could be smaller than the published intervention effect.

This ambiguity led the working group to suggest a wider range of plausible values for the intervention’s effect on severe hypoglycemia in the local setting, with their most realistic estimate representing less of an effect than the published estimate (elicited RR: 0.50, range: 0.20–0.75; published RR: 0.31; Table 3).

In the second elicitation session, the experts discussed scenarios to clarify their rationale for a reduced effect size in the local setting. It was agreed that the lower baseline event rate in the local setting could indicate that local patients experiencing hypoglycemia were less complex than patients experiencing hypoglycemia at the evaluation hospital. This lower complexity was interpreted to mean the intervention would be less effective in the local setting as it would be more challenging to address the highly variable causes of hypoglycemia in these lower-complexity patients (i.e., the experts disagreed with the presented scenarios’ assumption that hypoglycemia events would be easier to prevent in less complex patients). In addition, a more complex patient may receive more benefit from a review in which multiple potential contributors to hypoglycemia could be addressed, while a review of a less complex patient may provide less opportunity to make a large difference. Following discussion of the scenarios in session 2, the experts did not choose to adjust the intervention effect estimates provided for the local setting in session 1.

### Intervention Option 2: Root-Cause Survey with Targeted Education

For the root-cause survey, the experts concluded that from the limited information available, they did not expect quality of care in the local setting to be better or worse than at the study hospital. Thus, the lower event rates observed in the local setting suggested that patient complexity would be higher at the study sites. Following the same reasoning as discussed for the vGMS intervention, they expected the intervention to be less effective the local setting.

Most of the discussion focused on differences due to the scope of implementation. The experts observed the following:

- Patient complexity and clinician buy-in would differ between the study and local sites as the published evaluation included only general medical patients from 2 wards, and the intention was to implement the intervention hospital-wide in the local setting.
- The intervention may be less effective in surgical patients, in that patients’ nutritional intake can vary greatly day by day, compared with medical patients, in whom nutritional intake (even if poor) is likely to be relatively consistent over many days.
- Wider implementation was likely to reduce buy-in from clinical staff, compared with implementation in 1 or 2 wards where ownership of the intervention was likely to be higher and a culture of partnership and collaboration between nurses, junior doctors, and senior doctors regarding glycemic management could be nurtured. Without this partnership, there was likely to be a disconnect between the nurses who complete the survey and identify the cause of hypoglycemia and the clinical staff who have the capacity to adjust the patients’ glycemic management (e.g., insulin doses).

Given these concerns, the experts thought the intervention would be less effective in the local context compared with the study context. They estimated the intervention effect on hypoglycemia in the local setting to be a RR of 0.85 (range: 0.80–0.90), compared with the published RR of 0.68 (Table 3).

The published evaluation did not provide a separate estimate of the intervention effect for severe hypoglycemia. The working group observed that severe events are less predictable and therefore less preventable than nonsevere events are. However, based on the intervention characteristics, the group thought the intervention would most likely prevent severe hypoglycemia at the same rate as it prevented hypoglycemia in the local setting (RR: 0.85, range 0.80–0.90).

## Discussion

This article has reported on the use of expert elicitation methods to estimate the expected effects of multiple intervention options for preventing hypoglycemic events in the hospital, from the perspective of a specific local health service. Intervention options to be evaluated in a local-level economic evaluation were identified by undertaking a pragmatic literature review. However, the interventions were evaluated in quite different geographical and jurisdictional settings to the local health service for which a local-level economic evaluation was being undertaken. It was considered inappropriate to make an unassessed assumption that the interventions would have the same effects across different health service settings. Thus, a formal elicitation process was undertaken to assess and potentially adjust the published estimates of the interventions’ effects to reflect their expected effects in the local health service within which the local-level economic evaluation was being undertaken.

### Elicited Effect Estimates

The experts estimated lower expected intervention effects in the local setting. The “most optimistic” estimate for the effect of the vGMS intervention on severe hypoglycemia was the only elicited intervention effect that was greater than the published estimate (most optimistic RR: 0.20; published RR: 0.31); however, the wide elicited effect range indicated a large degree of uncertainty regarding the interventions effect in the local setting (RR range: 0.20–0.75).

For the vGMS intervention, the reduced effect elicited for the local setting was based on differences in patient complexity. The experts expected that the less complex patients seen in the local setting would make the intervention less effective in the local setting than in the published evaluation.

The reduced effect elicited for the root-cause survey intervention was largely driven by differences in the scope of implementation. Wider implementation in the local setting was likely to reduce buy-in from clinical staff, compared with the targeted implementation used in the evaluation (limited to 2 general medical wards). The experts from the working group identified this as something that required further consideration during any local implementation activities, for example, considering whether a staged implementation (rather than a single hospital-wide rollout) would mitigate this effect.

### The Elicitation Process

The elicitation process was undertaken with a multidisciplinary group of clinicians working in the local health service of interest, including clinicians with backgrounds relevant to the focus of the evaluation (hypoglycemia). Evidence to inform 3 broad areas of potential differences between the evaluation sites and the local health service were sought and presented to the experts: quality of care, patient complexity, and potential biases associated with the research study design and application. The evidence was prepared by 2 academic health economists who also facilitated the 2 elicitation sessions. Overall, the elicitation process was successful in facilitating the experts to consider the contextual information and provide effect estimates adjusted for the local setting.

The Bojke et al.^2,3^ reference protocol was chosen to guide the elicitation process as it provides a comprehensive overview of structured elicitation methods. Rather than specifying a single method, the Bojke et al.^2,3^ protocol summarizes the many ways elicitation has been undertaken and clearly sets out the choices to be made at each step in the process. It includes reference to the commonly used SHELF protocol (which elicits estimates from individual experts before using behavioral aggregation [group consensus] or mathematical aggregation [using the SHELF R package] to generate a single distribution) and the IDEA protocol (which elicits individual estimates from experts before combining them through quantile [mathematical] aggregation to generate a lower, best, and upper estimate).^2,3,13,14^

In the context of a working group that was established at the commencement of the local-level economic evaluation project, the most pragmatic and intuitive method was to undertake elicitation as a group, rather than with each individual separately, to elicit consensus effect estimates. The Bojke et al. protocol discusses group elicitation and the elements to be considered with this method. During the group elicitation process for this project, different members of the group contributed different perspectives, ideas, and interpretations to the discussion that legitimately informed the views of group members. The group discussed and debated until consensus values were agreed. Given the range of potential factors affecting the intervention effects in the local context, this discussion and debate increased the likelihood that the elicited values reflected all relevant factors. Involving the working group as the experts, undertaking elicitation as a group, and seeking a consensus estimate were important elements in ensuring the elicitation process could be completed in a timely and efficient manner.

Relative risks were elicited for each intervention for both hypoglycemia and severe hypoglycemia outcomes. This quantity was understood by the experts, and they were able to adjust it by drawing on their clinical insight and experience. Given relative risks were to be used in the subsequent local economic evaluation, it was the most appropriate quantity to elicit in this instance.

The expert group was asked to provide an upper, lower, and best (most realistic) estimate of the intervention’s effects. This pragmatic approach was taken to minimize the time required for elicitation (for example, in comparison with eliciting a distribution for each effect). It was sufficient, given the subsequent economic analysis did not intend to use probabilistic sensitivity analysis where a distribution would be required for each parameter. The definition of “best” was not predefined or discussed with the experts. Therefore, the estimates should not be used to generate a probability distribution in the future as it is unclear whether the “best” estimate would represent the mode, median, or mean.^13^

A key learning from the applied process was that the experts used the estimated baseline event rates as a focal point. They then used the available information on the 3 broad areas to try to explain the difference in baseline event rates between the 2 settings. The derived explanation then informed their estimates of expected intervention effects in the local context. The experts focused on contextual information regarding the described patient population, current models of care (e.g., glycemia management targets), and hospital characteristics such as designation, size, and catchment area. While they initially discussed differences in funding models between the United States and Australia, the experts did not dwell on this, and the facilitators did not probe further on this issue.

For each intervention, a 1-h meeting was scheduled for the experts to discuss the information provided and decide on the locally adjusted effect estimates for both hypoglycemia and severe-hypoglycemia outcomes. The second intervention took less time than the first, suggesting elicitation for subsequent interventions may be completed in about 40 min, although this will depend on the quantity and complexity of the information to be presented. Given the established relationships and familiarity in working together, both in-person and online formats worked well.

Both interventions for which elicitation was undertaken were evaluated in single hospitals using observational study designs. Had the studies been undertaken across multiple hospitals or via a randomized controlled trial, then more robust estimates could have been used as a starting point for elicitation, but potential differences between the study setting and local setting should still be considered to have confidence that the effect estimates are locally relevant.

From the working group’s clinical perspective, the elicitation process was a valuable activity to undertake as part of the local-level economic evaluation process. It offered the group the opportunity to reflect deeply on the interventions, consider the impact of differences in the evaluation and local contexts, and identify potential challenges regarding implementation, all of which allowed them to set realistic expectations of what the intervention may achieve if implemented in the local setting. Adjusting the published estimates for the local setting increased both the relevance of and the group’s comfort with the results of the subsequent cost-consequence analysis. It was seen to strengthen any future business case(s) submitted to the executive, as local validation had been undertaken. None of the expert group were familiar with elicitation; therefore, additional orientation materials describing the purpose and broad steps of the process would have been useful to include.

### Limitations

The capacity to compare the evaluation and local settings was limited by a lack of data, for example, where the published evaluation provided limited description of the baseline models of care from which information on quality of care could be extracted or where baseline patient characteristics were not well described in the evaluation. This required supplementation with externally sourced data broadly describing the evaluation hospital setting (e.g., from the study hospital Web sites). While general information on each hospital was easily available on their Web sites, the information was current as at the time of the elicitation (not from the time of the evaluation). Detailed information on the quality of care at the time of the study was available only in the published evaluation.

During the elicitation, the research team noted that biases from patient recruitment and data collection methods (see Tables 1 and 2) were relatively low due to the nonrecruitment of individual patients (the interventions were applied to all admitted patients) and the use of routinely collected, objective data to measure outcomes. The main risk of bias was perhaps regression to the mean if interventions were implemented in response to an increase in adverse outcomes; however, this limitation was not discussed with the experts.

The working group comprised local clinical stakeholders involved in the management of patients at risk of hypoglycemia, who had been given responsibility for recommending interventions to reduce hospital-acquired hypoglycemia rates. The group may be perceived to have a conflict of interest that could result in upwardly biased estimates of the elicited intervention effectiveness. However, throughout the elicitation process, the group was emphatic that they did not want to overestimate the expected intervention effects, resulting in, if anything, conservative elicited estimates of effect.

### Use of Elicitation for Local Economic Evaluations

In the context of health technology assessment, expert elicitation has mainly been used to estimate unknown or uncertain parameters for use in decision models.^2^ While advocating for the use of expert elicitation in the context of local decision making, Bojke et al.^2^ were unable to identify any documented examples of use in this context when developing their reference protocol. Economic evaluation itself is not routinely undertaken to inform decision making in local health services.^15^ Lack of relevance of published economic evaluations has been cited as a barrier to their use by local decision makers, as has a lack of experience in the use of economic evaluations to inform decisions.^16,17^ Both of these barriers may be at least partially addressed through the use of formal elicitation processes that support the interpretation and adjustment of evidence to better reflect expected intervention effects in the local context. The evaluation reported in this article provides evidence that a pragmatic elicitation process can allow meaningful adjustments to be elicited from local clinical experts who have no prior experience in the conduct or use of economic evaluation or elicitation processes.

## Conclusions

The pragmatic elicitation methods reported in this article provided a feasible and acceptable approach to assess and adjust published intervention effects to better reflect expected intervention effects in the local context. The further development and application of these methods are proposed as potentially important facilitators to the use of local economic evaluation.

## Supplemental Material

sj-pdf-1-mpp-10.1177_23814683231226335 – Supplemental material for Using Expert Elicitation to Adjust Published Intervention Effects to Reflect the Local Context Click here for additional data file. Supplemental material, sj-pdf-1-mpp-10.1177_23814683231226335 for Using Expert Elicitation to Adjust Published Intervention Effects to Reflect the Local Context by Jodi Gray, Tilenka R. Thynne, Vaughn Eaton, Rebecca Larcombe, Mahsa Tantiongco and Jonathan Karnon in MDM Policy & Practice
