# Supplementary material for: Using Expert Elicitation to Adjust Published Intervention Effects to Reflect the Local Context
Source: MDM Policy Pract. 2024 Jan 25;9(1):23814683231226335. doi: 10.1177/23814683231226335 (PMC10812103; doi:10.1177/23814683231226335)
Supplement: sj-pdf-1-mpp-10.1177_23814683231226335 – Supplemental material for Using Expert Elicitation to Adjust Published Intervention Effects to Reflect the Local Context [file sj-pdf-1-mpp-10.1177_23814683231226335.pdf]

Using expert elicitation to adjust published intervention effects to reflect the local context

Supplementary Materials A: Elicitation PowerPoint Slides

Session 1

Intervention 1:
Virtual Glycaemic Management Service (vGMS)

Why elicitation?

- The published effect estimates for the interventions will be influenced by the **context** in which they were evaluated:
  - Patient characteristics
  - Quality of care in the hospital
  - Research context
- Use **your clinical expertise and knowledge** to:
  - Consider the differences between the study context and FMC
  - Estimate how these differences may modify the effectiveness of the intervention

vGMS (Rushakoff 2017)

Introduction to elicitation and what we were going to ask the experts to do during the session(s).

Note: The 'Research context' is referred to as 'potential biases associated with the research study design and application' in the main manuscript.

Virtual Glycaemic Management Service (vGMS)

The intervention involves:

- Daily automated report of potentially ‘at risk’ patients generated via the EMR
- At risk is defined as having in the previous 24 hours:
  - One or more BGLs <70 mg/dL (3.9 mmol/L)
  - OR two or more BGLs ≥225 mg/dL (12.5 mmol/L)
  - OR on an insulin pump
- The report is reviewed by the vGMS team prior to morning rounds (endocrinologist, diabetes educator nurse, or diabetes educator pharmacist)
- Notes are added to the patients’ EMR suggesting changes to glycaemic management if required.

vGMS (Rushakoff 2017)

Reminder of what the intervention involves.

Differences between the study hospital & FMC…

vGMS (Rushakoff 2017)

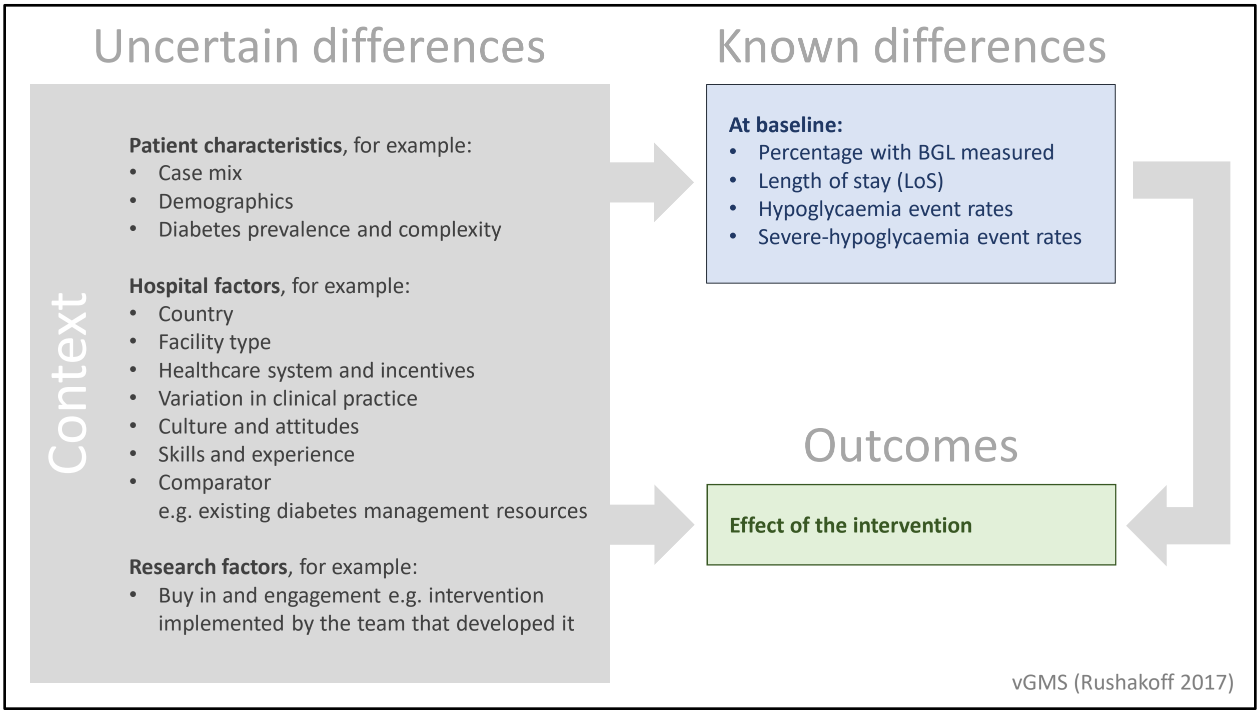

Conceptual overview of the differences in the study and local settings that may impact the effect estimate.
Known differences are where quantitative measures were available for both the study and local settings.

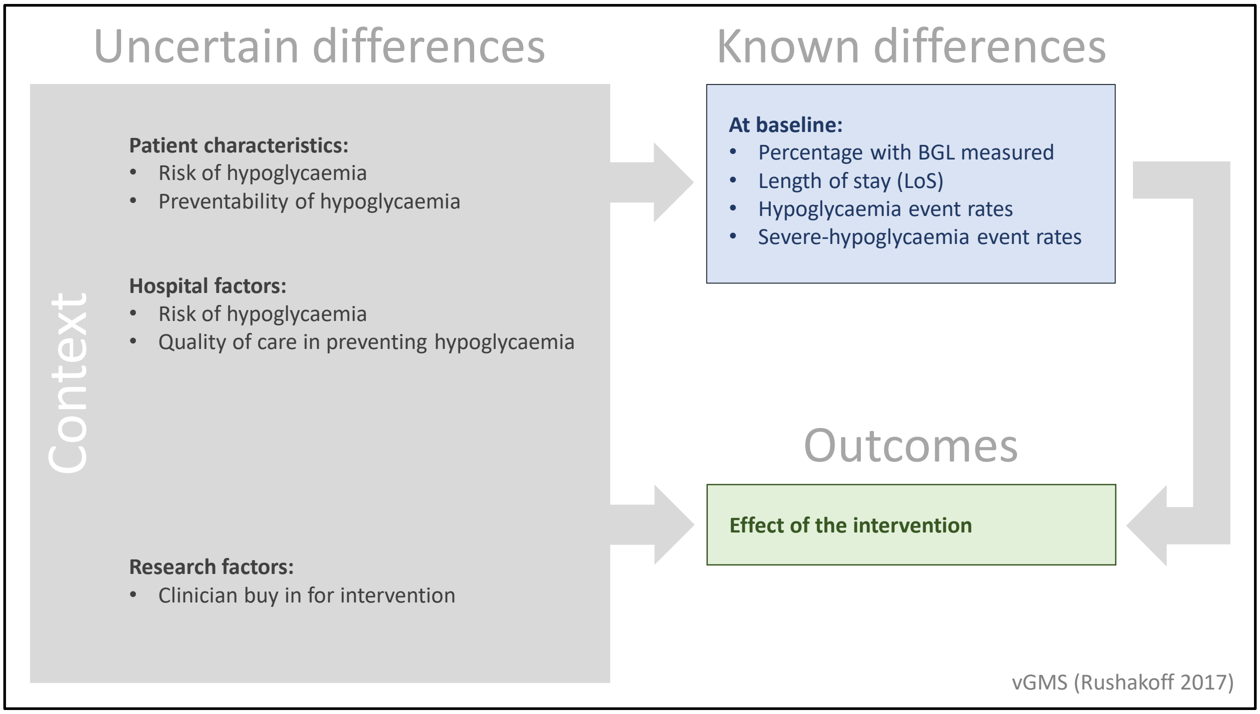

Summarising the uncertain differences to highlight the key means by which they may influence the effect of the intervention.

Patient characteristics

In general:

- San Francisco, USA
- Data from 2012-15
- Hospital types: Quarternary care (QC), Academic, and Veterans Affairs

- UCSF Health:
  - QC: highest designation for facilities that treat the most complex and specialized conditions
  - Provide more hospital care for Medicaid patients than any other hospital in San Francisco

Study specific:

	Study hospital	FMC	Relative differences
Percentage with a POC-BGL measured:	29%	26%	12% higher
Mean length of stay (days):	8.4	4.3	2x higher
Hypoglycaemia rate (per 100 patient days):	0.78	0.53	47% higher
Severe-hypoglycaemia rate (per 100 patient days):	0.032	0.054	40% lower

Additional notes on patient characteristics and setting (which were to be mentioned if required):

- Medicaid is the largest source of funding for medical and health-related services for people with low income in the United States
- Study patient cohort was: All non-obstetric adult patients (with / without PoC-BGLs)
- Study BGL targets were: 3.9-10.0 mmol/L (70-180 mg/dL)

Patient characteristics

In general:

- San Francisco, USA
- Data from 2012-15
- Hospital types: Quarternary care (QC), Academic, and Veterans Affairs

- UCSF Health:
  - QC: highest designation for facilities that treat the most complex and specialized conditions
  - Provide more hospital care for Medicaid patients than any other hospital in San Francisco

Are the study hospital patients more, less or the same complexity as FMC patients?

Study specific:

	Study hospital	FMC	Relative differences
Percentage with a POC-BGL measured:	29%	26%	12% higher
Mean length of stay (days):	8.4	4.3	2x higher
Hypoglycaemia rate (per 100 patient days):	0.78	0.53	47% higher
Severe-hypoglycaemia rate (per 100 patient days):	0.032	0.054	40% lower

'Complexity' refers to the likelihood that the patient will experience hypoglycaemia.

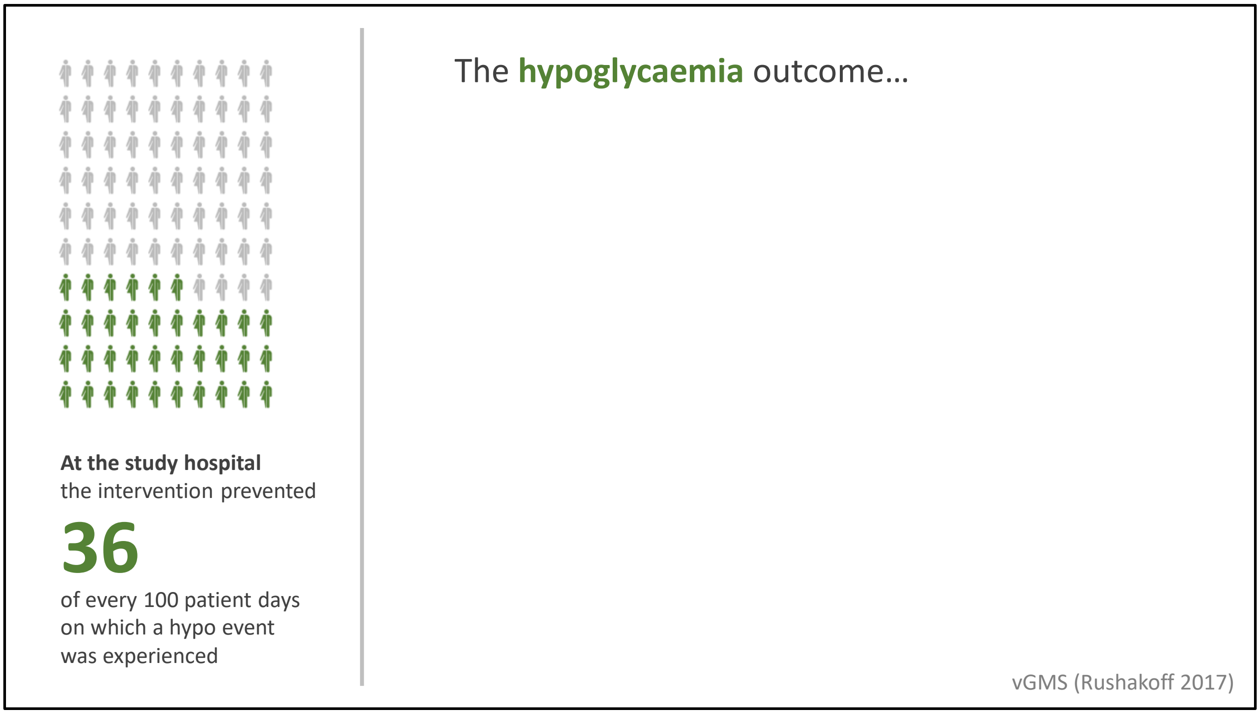

RR: 0.64 (95% CI: 0.57, 0.70), p<0.001
Translates to: 36 (95% CI: 30 to 43) prevented patient days out of 100 patients days with an event

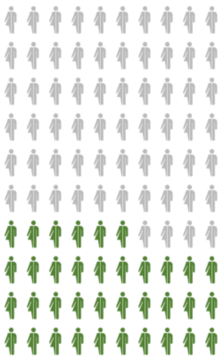

At the study hospital
the intervention prevented

36

of every 100 patient days
on which a hypo event
was experienced

Thinking about the **complexity of patients**
at FMC compared to the study hospital…

At FMC
do you think the intervention would prevent

more, less or the same

out of every 100 patient days
on which a hypo event was experienced?

Hospital characteristics

In general:

- San Francisco, USA
- Data from 2012-15
- Hospital types: Quarternary care, Academic, and Veterans Affairs

- UCSF Health:
  - Ranked #1 hospital in California and #6 in USA
  - Ranked in top 10 US hospitals for diabetes and endocrinology care

Study specific (baseline care):

Infrastructure:
- **Diabetes committee** provided oversight
- **EMR** newly implemented in months before baseline
- Computerised insulin **orders sets** (for eating, NPO, IV)
- **PoC-BGLs** automatically uploaded to EMR in real time
- **BGL tests** were done:
  - 5x per day if eating
  - 6x per day if NPO, total parenteral nutrition, or enteral feedings
  - Hourly if IV insulin infusion

Personnel:
- **Experienced staff** (endocrinologists, CDE nurses, CDE pharmacists) in managing inpatient diabetes
- Medical, nursing and pharmacy staff were **well-trained** (insulin pharmacodynamics and inpatient insulin regimens) and **receptive** to recommendations

Additional notes on patient characteristics and setting (which were to be mentioned if required):
- Medicaid is the largest source of funding for medical and health-related services for people with low income in the United States
- Study BGL targets were: 3.9-10.0 mmol/L (70-180 mg/dL)

Hospital characteristics

In general:

- San Francisco, USA
- Data from 2012-15
- Hospital types: Quarternary care, Academic, and Veterans Affairs

- UCSF Health:
  - Ranked #1 hospital in California and #6 in USA
  - Ranked in top 10 US hospitals for diabetes and endocrinology care

Is the quality of care at the study hospital more, less or the same as at FMC?

Study specific (baseline care):

Infrastructure:

- **Diabetes committee** provided oversight
- **EMR** newly implemented in months before baseline
- Computerised insulin **orders sets** (for eating, NPO, IV)
- **PoC-BGLs** automatically uploaded to EMR in real time
- **BGL tests** were done:
  - 5x per day if eating
  - 6x per day if NPO, total parenteral nutrition, or enteral feedings
  - Hourly if IV insulin infusion

Personnel:

- **Experienced staff** (endocrinologists, CDE nurses, CDE pharmacists) for managing inpatient diabetes
- Medical, nursing and pharmacy staff were **well-trained** (insulin pharmacodynamics and inpatient insulin regimens) and **receptive** to recommendations

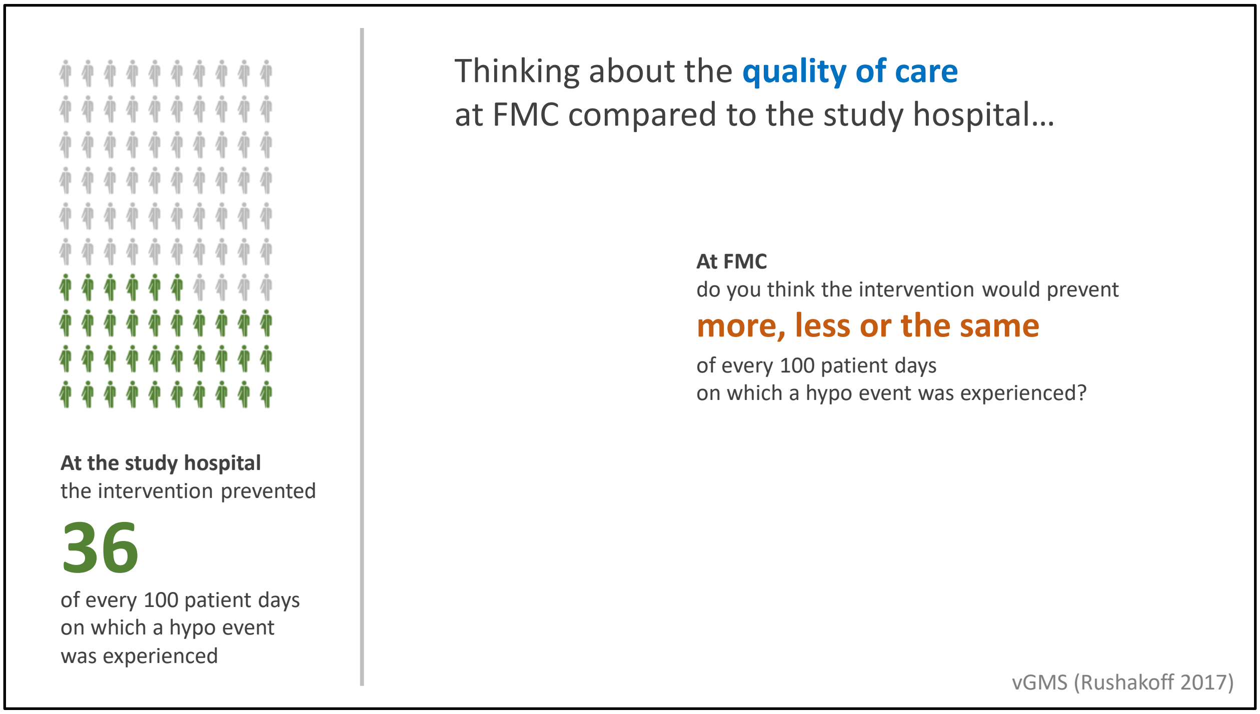
Thinking about the quality of care
at FMC compared to the study hospital…
At the study hospital
the intervention prevented
36
of every 100 patient days
on which a hypo event
was experienced
At FMC
do you think the intervention would prevent
more, less or the same
of every 100 patient days
on which a hypo event was experienced?
vGMS (Rushakoff 2017)

Research context

Study specific:

- Observational study design
- Included all adult patients (exc. obstetrics)
- Used routinely collected data from the EMR

- **Intervention was designed and implemented by clinicians at the hospital**
  - Therefore may have greater buy in from the vGMS team, endocrinology, management and hospital staff in general.

vGMS (Rushakoff 2017)

Note: The 'Research context' is referred to as 'potential biases associated with the research study design and application' in the main manuscript.

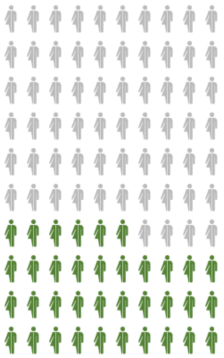

At the study hospital
the intervention prevented

36

of every 100 patient days
on which a hypo event
was experienced

Thinking about the **research context**
at the study hospital…

At FMC
do you think the intervention would prevent
more, less or the same
of every 100 patient days
on which a hypo event was experienced?

vGMS (Rushakoff 2017)

In summary, you thought that…

Patient complexity was

higher / lower at FMC
compared to the study hospital.

Which meant at FMC
the intervention would prevent

more / less / the same
out of every 100 patient days
on which a hypo event
was experienced.

Quality of care was

higher / lower at FMC
compared to the study hospital.

Which meant at FMC
the intervention would prevent

more / less / the same
out of every 100 patient days
on which a hypo event
was experienced.

The research context was

the same / different at FMC
compared to the study hospital.

Which meant at FMC
the intervention would prevent

more / less / the same
out of every 100 patient days
on which a hypo event
was experienced.

vGMS (Rushakoff 2017)

Slide was to be edited during the session to summarise the discussion.

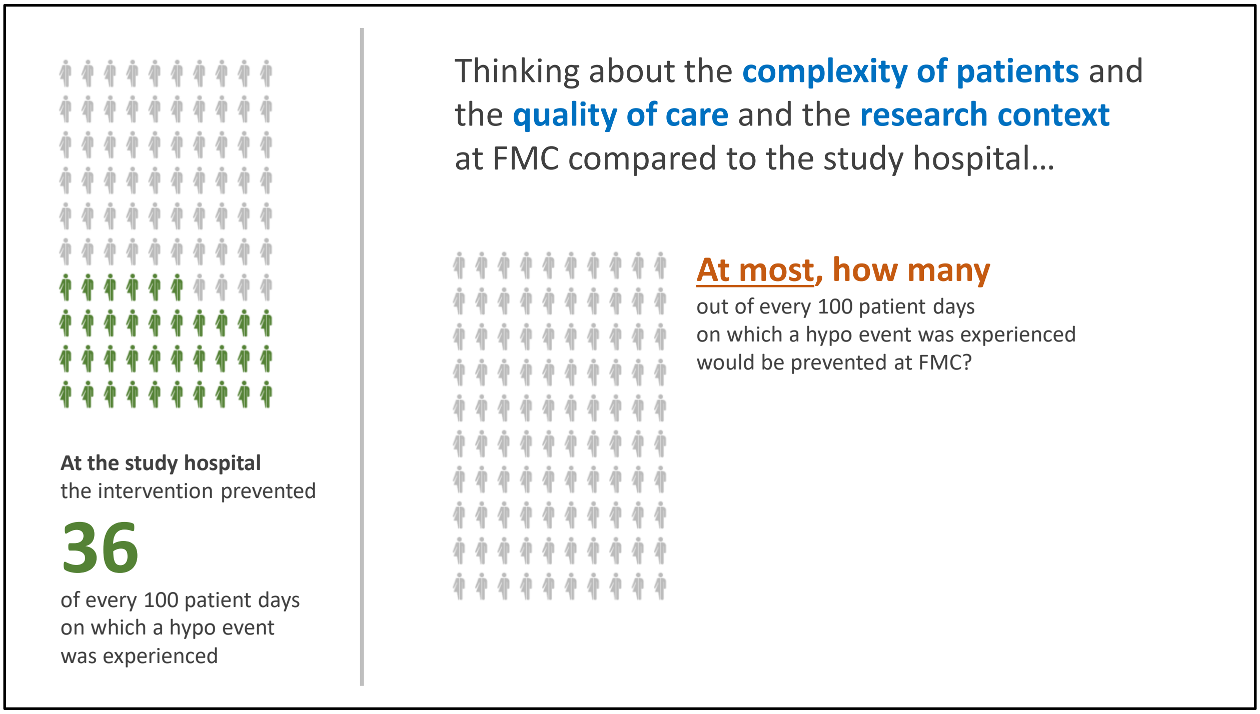
At the study hospital
the intervention prevented
36
of every 100 patient days
on which a hypo event
was experienced
Thinking about the complexity of patients and
the quality of care and the research context
at FMC compared to the study hospital…
At most, how many
out of every 100 patient days
on which a hypo event was experienced
would be prevented at FMC?

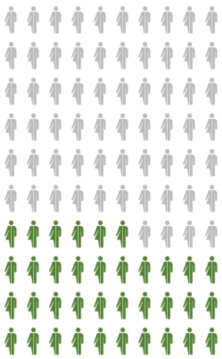

At the study hospital
the intervention prevented
36
of every 100 patient days
on which a hypo event
was experienced

Thinking about the **complexity of patients** and the **quality of care** and the **research context** at FMC compared to the study hospital…

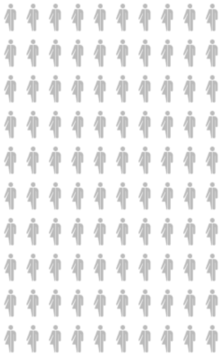

At least, how many
out of every 100 patient days
on which a hypo event was experienced
would be prevented at FMC?

vGMS (Rushakoff 2017)

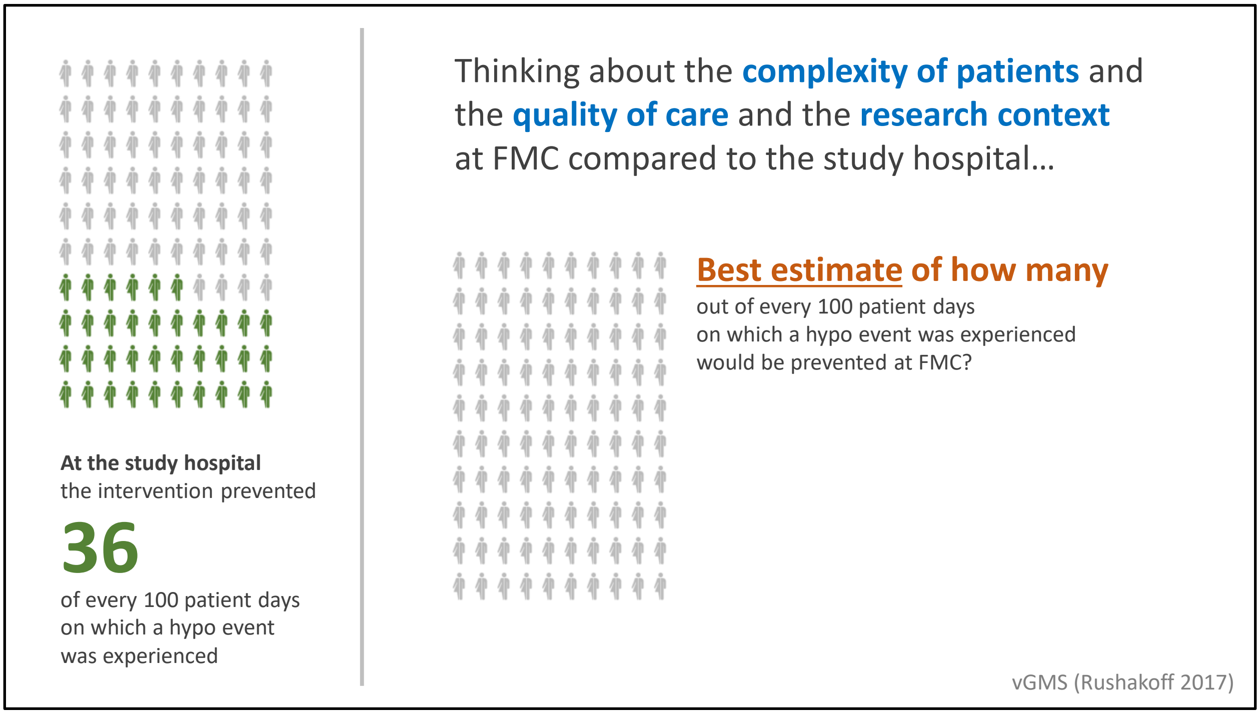
Thinking about the complexity of patients and the quality of care and the research context at FMC compared to the study hospital…
Best estimate of how many
out of every 100 patient days
on which a hypo event was experienced
would be prevented at FMC?
At the study hospital
the intervention prevented
36
of every 100 patient days
on which a hypo event
was experienced
vGMS (Rushakoff 2017)

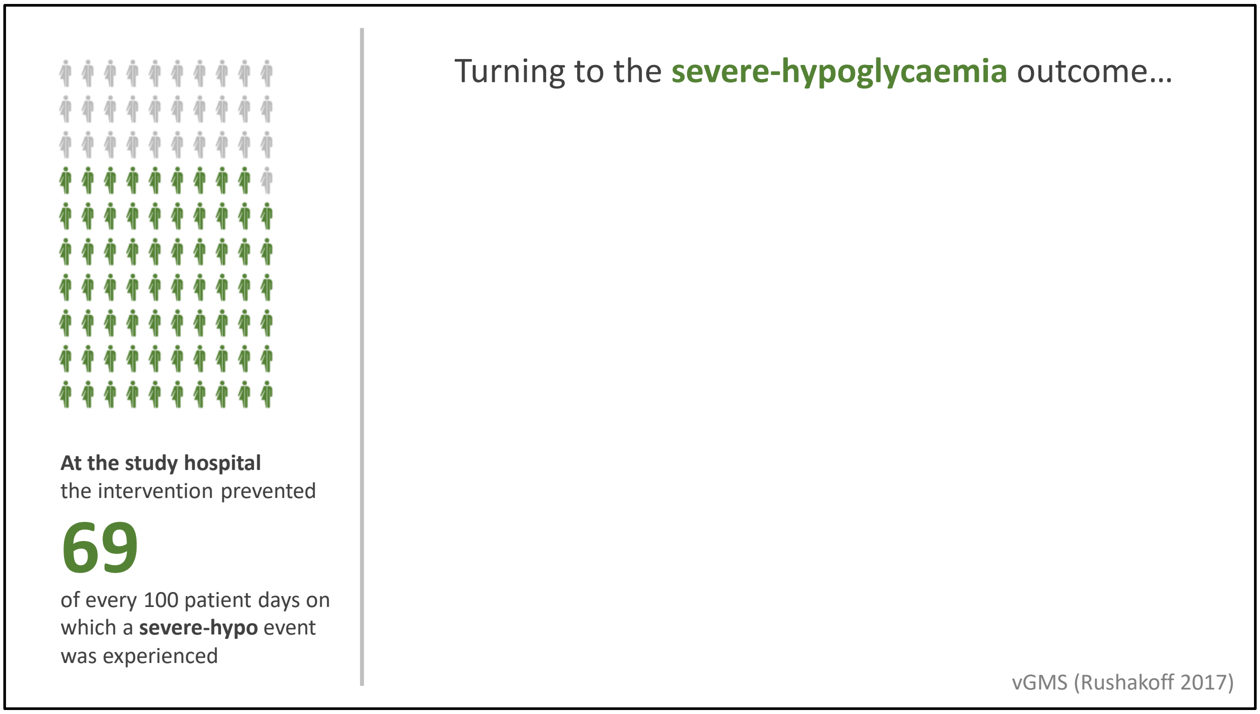

RR: 0.31 (CI: 0.15, 0.59), $p<0.001$
Translates to: 69 (95% CI: 41 to 85) prevented patient days out of 100 patients days with an event

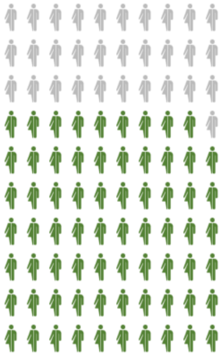

At the study hospital
the intervention prevented

69

of every 100 patient days on which a **severe-hypo** event was experienced

Thinking about the **complexity of patients** and the **quality of care** and the **research context** at FMC compared to the study hospital…

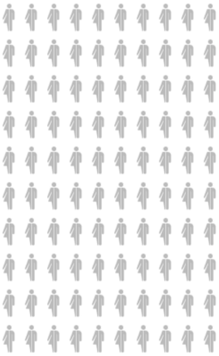

At most, how many
out of every 100 patient days
on which a **severe-hypo** event was experienced
would be prevented at FMC?

vGMS (Rushakoff 2017)

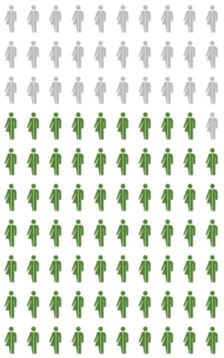

At the study hospital
the intervention prevented

69

of every 100 patient days on which a **severe-hypo** event was experienced

Thinking about the **complexity of patients** and the **quality of care** and the **research context** at FMC compared to the study hospital…

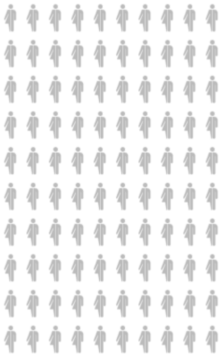

At least, how many
out of every 100 patient days
on which a **severe-hypo** event was experienced
would be prevented at FMC?

vGMS (Rushakoff 2017)

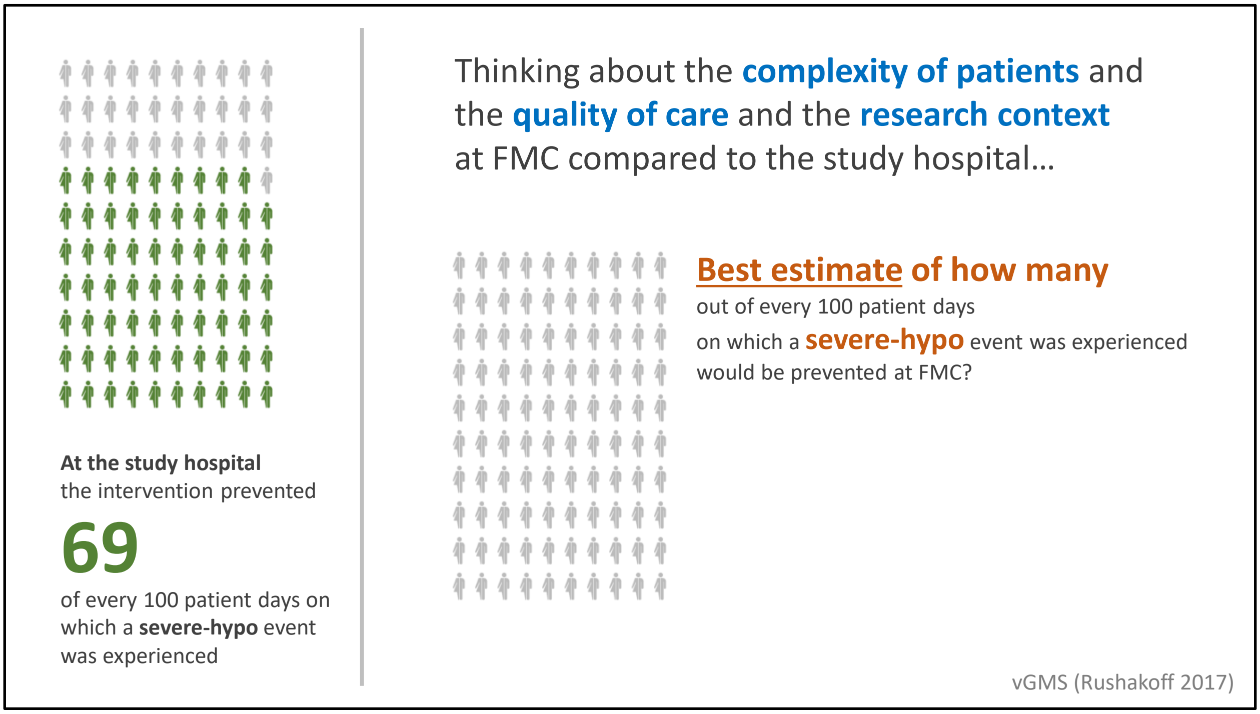
At the study hospital
the intervention prevented
69
of every 100 patient days on which a severe-hypo event was experienced
Thinking about the complexity of patients and the quality of care and the research context at FMC compared to the study hospital…
Best estimate of how many
out of every 100 patient days
on which a severe-hypo event was experienced
would be prevented at FMC?
vGMS (Rushakoff 2017)

Session 2

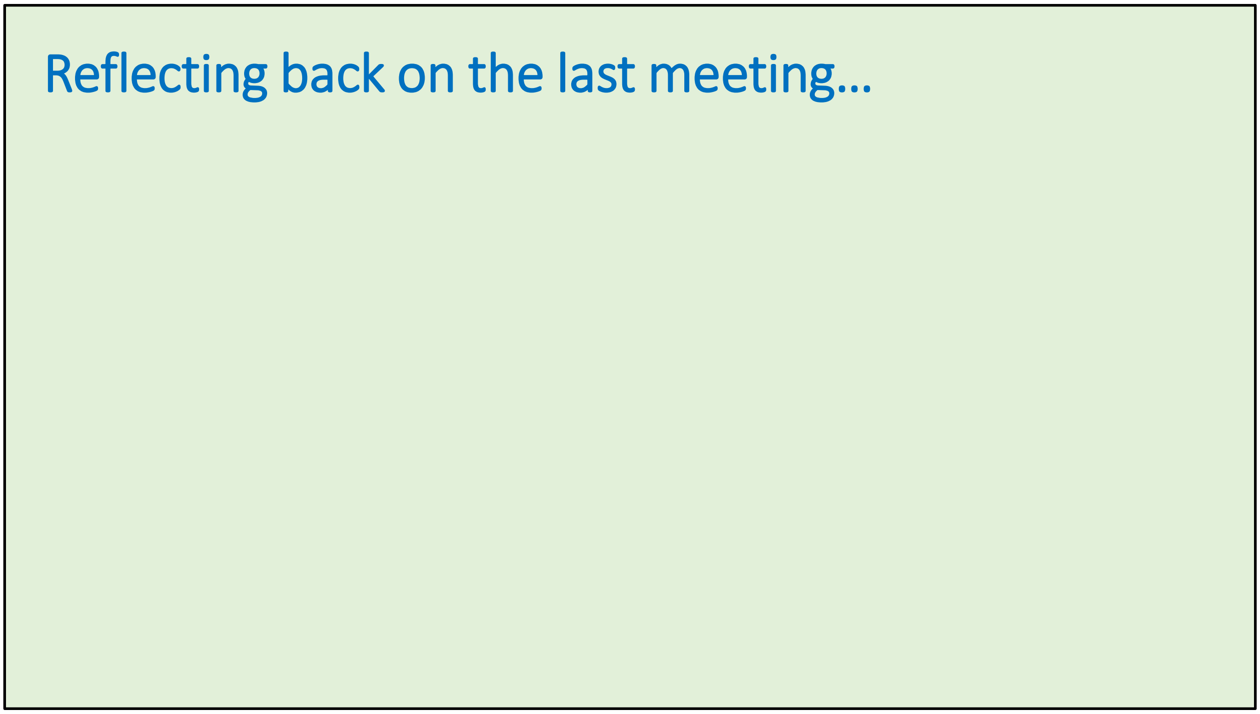

i.e. on the vGMS intervention

In summary, you thought that…

The research context was
the same at FMC
compared to the study hospital.

Which meant at FMC
the intervention would prevent
the same
out of every 100 patient days on which
a hypo event was experienced.

vGMS (Rushakoff 2017)

For all hypo events, you thought that…

Hypoglycaemia rate (per 100 patient days):

Study hospital	FMC
**0.78**	**0.53**

47% higher

In general, patient complexity and **quality of care** was the **same at FMC** compared to the study hospital.

However, lower baseline **hypo** rates at FMC were most likely due to some "differences in patient complexity".

Scenario (A)
"FMC had already addressed the 'low hanging fruit'" which suggests that at baseline

- FMC was better at **preventing hypo events in lower complexity** patients
- Therefore **more complex patients** were experiencing hypo events at FMC compared to the study hospital
- Therefore the intervention would **prevent a lower proportion** of hypo events in the more complex patients?

Alternatively, Scenario (B)
The difference in baseline hypo event rates is **because the study hospital sees more complex patients** (and not because FMC was better at preventing hypo events in lower complexity patients)

This suggests that

- Overall **less complex patients** were experiencing hypo events at FMC compared to the study hospital
- Therefore the intervention would **prevent a higher proportion** of hypo events in the less complex patients?

Which scenario do you think is most realistic?

These were the alternative scenarios relating to the relationship between quality of care and patient characteristics and the differences in hypoglycaemia event rates that were presented to the experts.

They were intended to clarify the researcher's understanding of the expert's discussion from session 1.

The scenarios made assumptions about how the complexity of patients related to the preventability of hypoglycaemic events – i.e. that events were easier to prevent in lower complexity patients.

The experts strongly disagreed with this assumption and rejected these scenarios. However, they did generate discussion which clarified the rationale for the effect estimates provided.

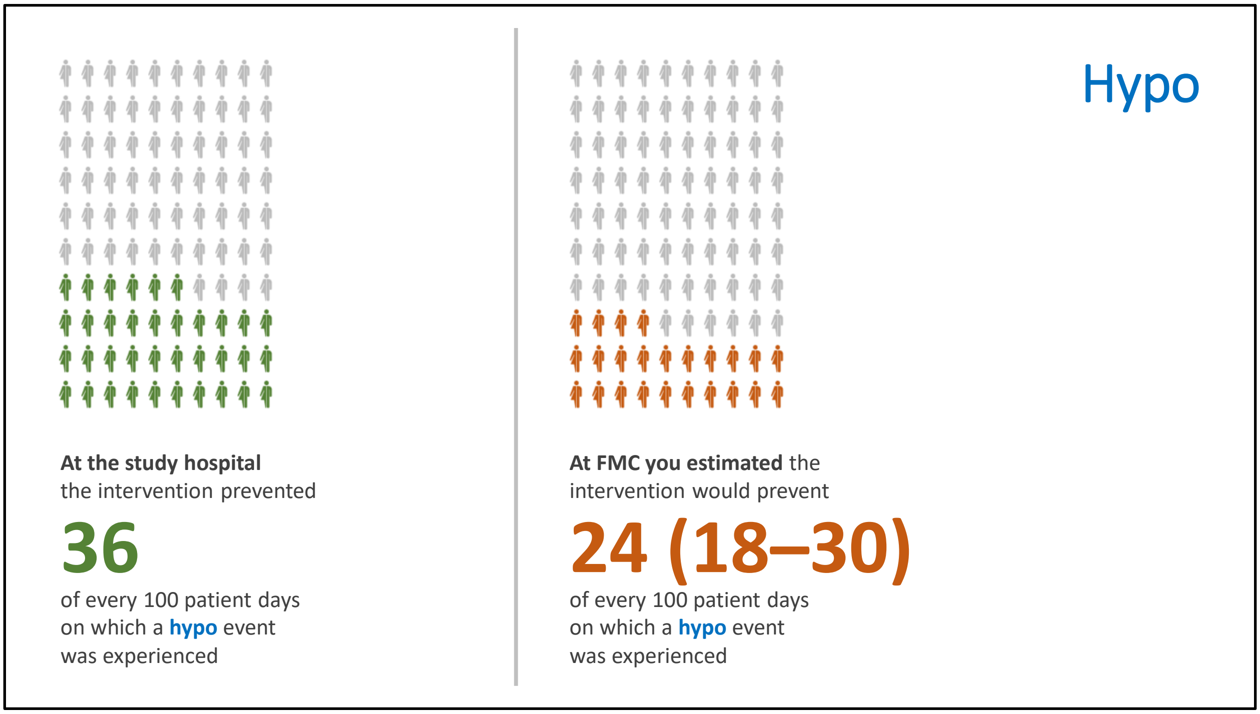

The published RR for the vGMS intervention compared to the RR elicited in the first session (with the range provided by the most optimistic and most pessimistic estimates).

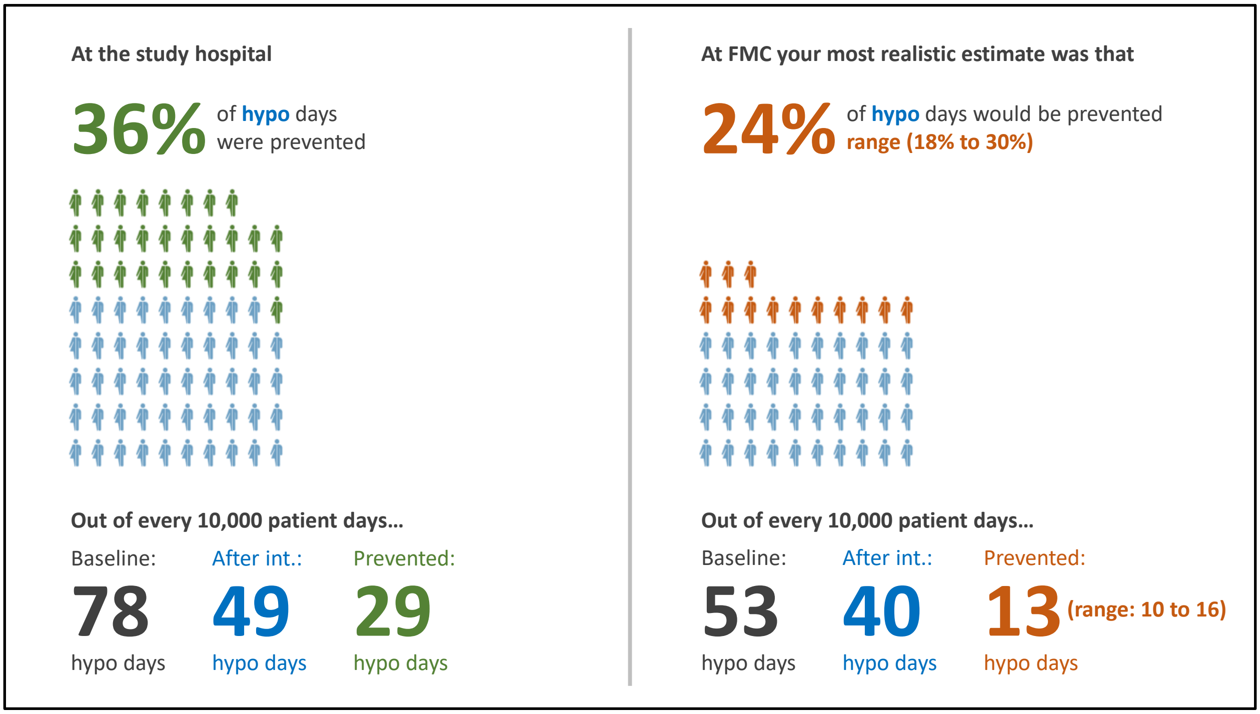

Translating the RRs into the number of patient days with hypoglycaemia.
Enables comparison of pre and post intervention numbers as well as number prevented between the study setting and predicted for the local (FMC) setting.
Aim was to ensure the difference between a relative effect (the RR) and an absolute effect (the number of patient days prevented) was clear.

Intervention 2:
Root cause survey and targeted education

Root cause survey + targeted education

The intervention involved:

- (Preliminary survey of nurses to shortlist causes of hypoglycaemia).
- Automated EMR survey of nursing staff for real-time identification of hypoglycaemic events and root causes.
- Clinical audit of hypoglycaemic events by a physician.
- Brief targeted education addressing the main cause identified using a 10 minute PowerPoint and a 1 page handout
  - Completed education on insulin dosing / titration (#1 cause).
  - Planned future education on interruptions in nutrition (#2 cause).
- Process engaged with and empowered nurses (and physicians) on hypoglycaemia prevention and glycaemic control.

Root cause survey (Sinha Gregory 2018)

Reminder of what is involved in the intervention.

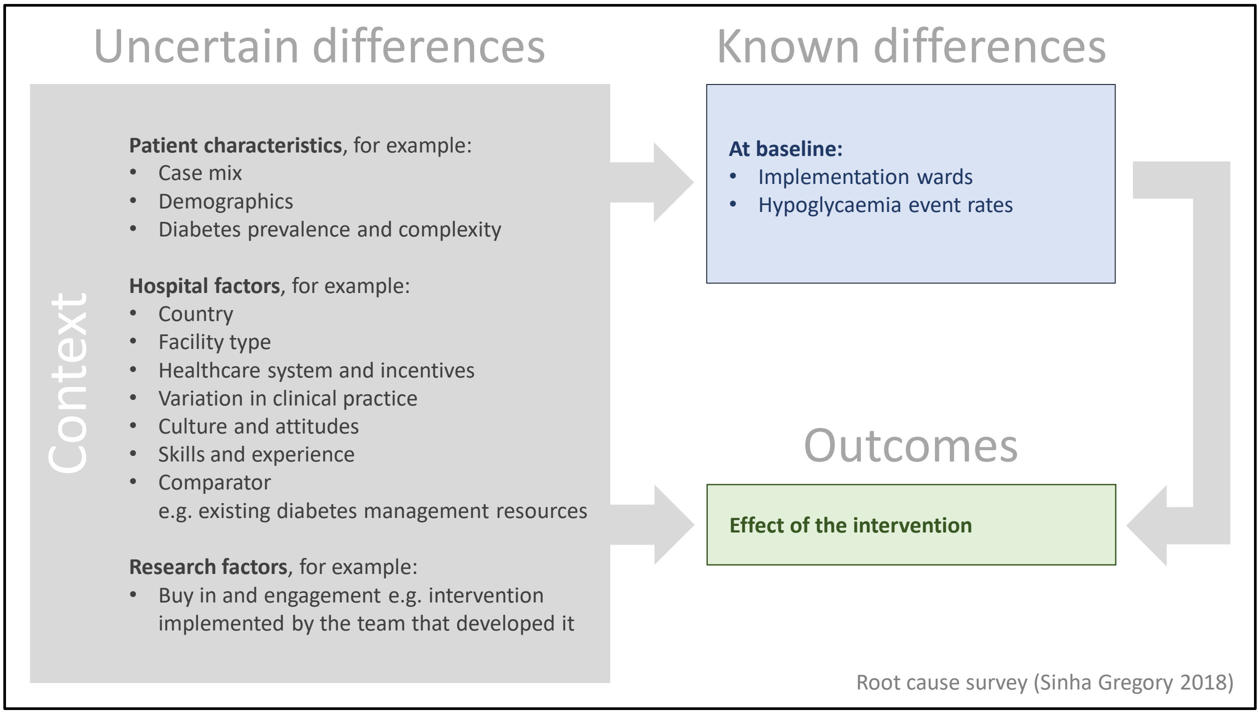

Conceptual overview of the differences in the study and local settings that may impact the effect estimate.
Known differences are where quantitative measures were available for both the study and local settings (differs between the two interventions).

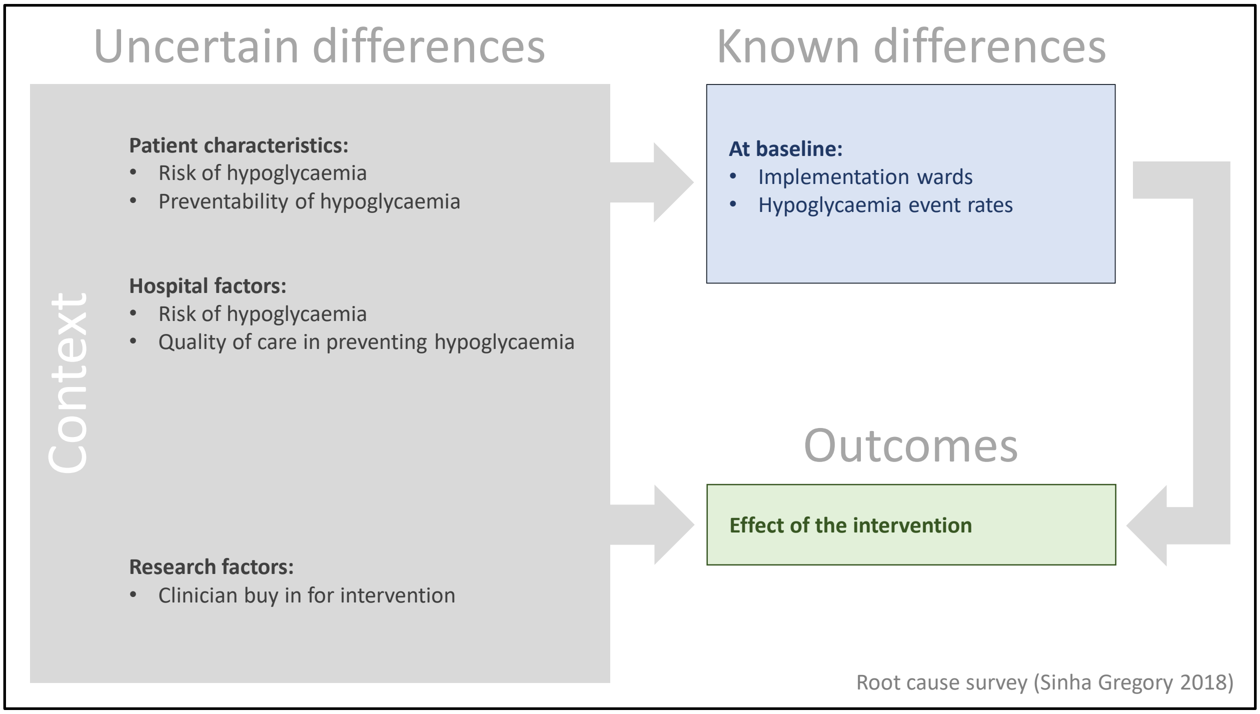

Summarising the uncertain differences to highlight the key means by which they may influence the effect of the intervention.

Hospital (and general patient) characteristics

In general:

- New York, USA
- Data from 2016-17
- Hospital type: Academic medical centre

- New York-Presbyterian: Weill Cornell Medical Center
    - Ranked #1 hospital in New York and #7 in USA
    - Ranked #5 in US for diabetes and endocrinology
    - National recognition for excellence in nursing (top 8% of US hospitals).

Baseline care:

Infrastructure:
- **EMR** (Sunrise)
- **PoC-BGLs** automatically uploaded to EMR in real time
- EMR included a **glycaemic control dashboard** showing BGLs and insulin dose in 4 time buckets (meals, overnight)

Presenting general characteristics for the hospital and patients together.

Hospital (and general patient) characteristics

In general:

- New York, USA
- Data from 2016-17
- Hospital type: Academic medical centre

- New York-Presbyterian: Weill Cornell Medical Center
  - Ranked #1 hospital in New York and #7 in USA
  - Ranked #5 in US for diabetes and endocrinology
  - National recognition for excellence in nursing (top 8% of US hospitals).

Are the study hospital patients more, less or the same complexity as FMC patients?

Baseline care:

Infrastructure:

- **EMR** (Sunrise)
- **PoC-BGLs** automatically uploaded to EMR in real time
- EMR included a **glycaemic control dashboard** showing BGLs and insulin dose in 4 time buckets (meals, overnight)

Is the quality of care at the study hospital more, less or the same as at FMC?

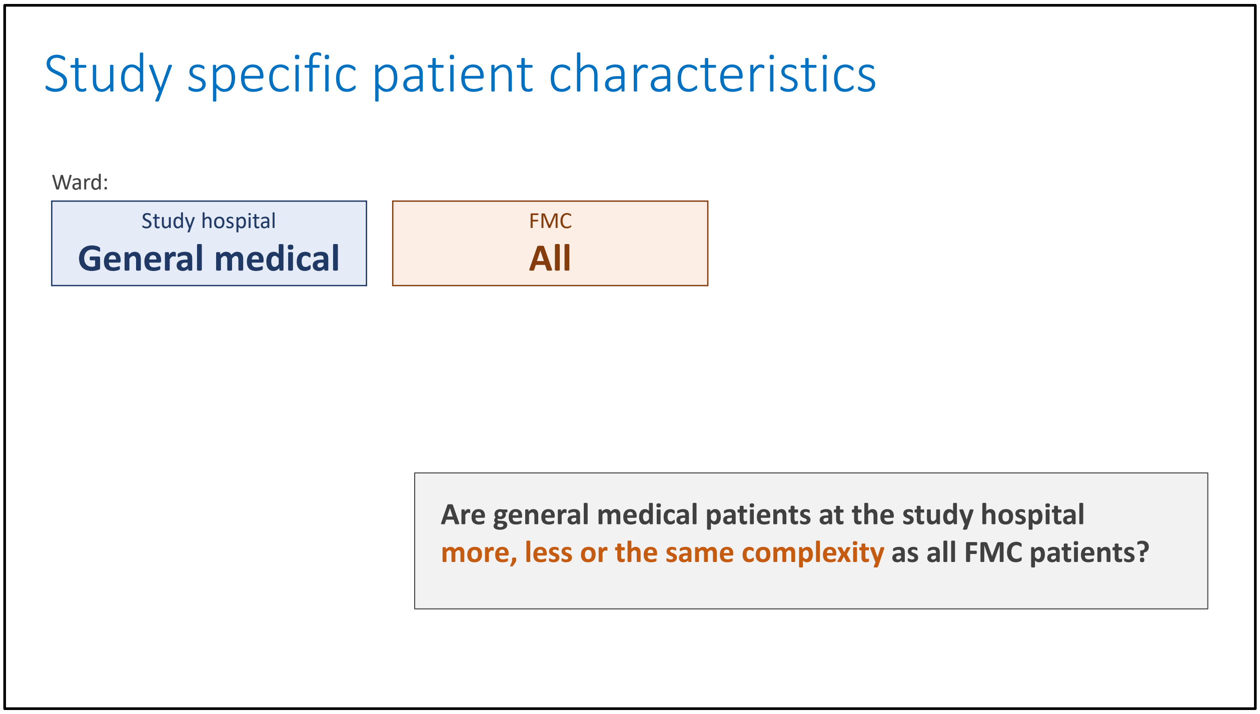

By complexity we were referring to the likelihood that the patient will experience hypoglycaemia.

Additional notes on patient characteristics and setting (which were to be mentioned if required):

- Study patient cohort: 2 general medical wards over a two month period.
- Study target BGLs: 3.9-10.0 mmol/L (70-180 mg/dL)

For all hypo events…

Hypoglycaemia rate (per 100 BGL measurements):

Study hospital	FMC
**2.27**	**1.17**

94% higher

Scenario (A)

"FMC had already addressed the 'low hanging fruit'" which suggests that at baseline

- FMC was better at **preventing hypo events in lower complexity** patients
- Therefore **more complex patients** were experiencing hypo events at FMC compared to the study hospital
- Therefore the intervention would **prevent a lower proportion** of hypo events in the more complex patients?

Scenario (B)

The difference in baseline hypo event rates is **because the study hospital sees more complex patients** (and not because FMC was better at preventing hypo events in lower complexity patients)

This suggests that

- Overall **less complex patients** were experiencing hypo events at FMC compared to the study hospital
- Therefore the intervention would **prevent a higher proportion** of hypo events in the less complex patients?

Scenario (C)

The difference in baseline hypo event rates is **because the study hospital included general medical patients only and FMC is hospital-wide** (all adult, exc. obstetrics).

- And the intervention would **prevent a more? less? same? proportion** of hypo events because… ?

These were the alternative scenarios relating to the relationship between quality of care and patient characteristics and the differences in hypoglycaemia event rates that were presented to the experts.

They were intended to clarify the researcher's understanding of the expert's discussion from session 1.

The scenarios made assumptions about how the complexity of patients related to the preventability of hypoglycaemic events – i.e. that events were easier to prevent in lower complexity patients.

The experts strongly disagreed with this assumption and rejected these scenarios.

During the discussions, Scenario B dot point two was changed from 'would prevent a HIGHER proportion of hypo events' to 'would prevent a LOWER proportion of hypo events' to more accurately represent the thinking of the experts.

For all hypo events…

Which scenario do you think is most realistic?

Hypoglycaemia rate (per 100 BGL measurements):

Study hospital	FMC	
**2.27**	**1.17**	94% higher

Scenario (A)
"FMC had already addressed the 'low hanging fruit'" which suggests that at baseline

- FMC was better at **preventing hypo events in lower complexity** patients
- Therefore **more complex patients** were experiencing hypo events at FMC compared to the study hospital
- Therefore the intervention would **prevent a lower proportion** of hypo events in the more complex patients?

Scenario (B)
The difference in baseline hypo event rates is **because the study hospital sees more complex patients** (and not because FMC was better at preventing hypo events in lower complexity patients)

This suggests that

- Overall **less complex patients** were experiencing hypo events at FMC compared to the study hospital
- Therefore the intervention would **prevent a higher proportion** of hypo events in the less complex patients?

Scenario (C)
The difference in baseline hypo event rates is **because the study hospital included general medical patients only and FMC is hospital-wide** (all adult, exc. obstetrics).

- And the intervention would **prevent more / less / the same proportion** of hypo events because… ?

These were the alternative scenarios relating to the relationship between quality of care and patient characteristics and the differences in hypoglycaemia event rates that were presented to the experts.

They were intended to clarify the researcher's understanding of the expert's discussion from session 1.

The scenarios made assumptions about how the complexity of patients related to the preventability of hypoglycaemic events – i.e. that events were easier to prevent in lower complexity patients.

The experts strongly disagreed with this assumption and rejected these scenarios.

During the discussions, Scenario B dot point two was changed from 'would prevent a HIGHER proportion of hypo events' to 'would prevent a LOWER proportion of hypo events' to more accurately represent the thinking of the experts.

The **hypoglycaemia** outcome…

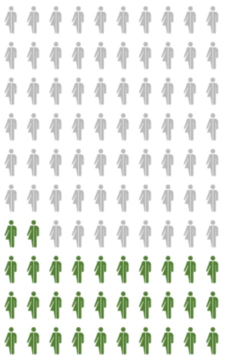

At the study hospital
the intervention prevented

32

of every 100 hypoglycaemic PoC-BGL measurements

Root cause survey (Sinha Gregory 2018)

RR: 0.68 (CI: 0.49, 0.94), p:0.019
Translates to: prevented 32 (95% CI: 6 to 51) hypoglycaemic PoC-BGL measures out of 100 hypoglycaemic PoC-BGL measures

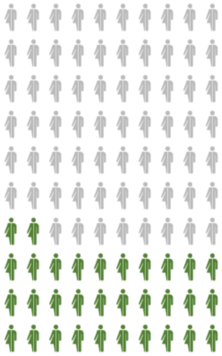

At the study hospital
the intervention prevented
32
of every 100 hypoglycaemic
PoC-BGL measurements

Thinking about the **complexity of patients** and **the quality of care** at FMC compared to the study hospital…

At FMC you thought
the intervention would prevent
more, less or the same
out of every
100 hypoglycaemic PoC-BGL measurements?

Research context

Study specific:

- Observational study design
- Included patients from two general medical wards
- Used routinely collected data from the EMR

- **Intervention was designed and implemented by clinicians at the hospital**
    - Therefore may have greater buy in from hospital staff and management.

Root cause survey (Sinha Gregory 2018)

Additional information:
- Study used 2 months of data

Note: The 'Research context' is referred to as 'potential biases associated with the research study design and application' in the main manuscript.

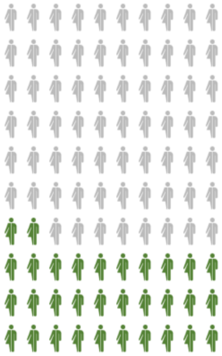

At the study hospital
the intervention prevented

32

of every 100 hypoglycaemic
PoC-BGL measurements

Thinking about the **research context**
at the study hospital…

At FMC
do you think the intervention would prevent

more, less or the same

out of every
100 hypoglycaemic PoC-BGL measurements?

Root cause survey (Sinha Gregory 2018)

In summary, you thought that…

The **difference in baseline event rates** for all hypo events was (94%) higher at the study hospital because:

A. FMC was better at preventing hypo events in less complex patients at baseline?
B. The study hospital sees more complex patients?
C. The study hospital included only general medical patients and FMC was hospital-wide?
D. A combination of the above?

And this would:

- Reduce the expected effect of the intervention at FMC
- Increase the expected effect of the intervention at FMC
- Have no impact on the expected effect of the intervention at FMC

The **research context** of the intervention's implementation at the study hospital would:

- Reduce the expected effect of the intervention at FMC
- Increase the expected effect of the intervention at FMC
- Have no impact on the expected effect of the intervention at FMC

Root cause survey (Sinha Gregory 2018)

Did not show this slide due to time constraints during session 2. The intention was to update the slide during the session based on the discussion.

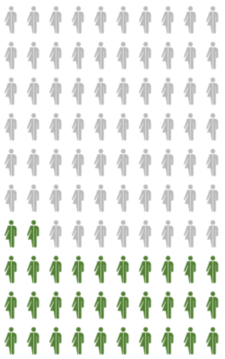

At the study hospital
the intervention prevented

32

of every 100 hypoglycaemic PoC-BGL measurements

Thinking about the **complexity of patients** and the **quality of care** and the **research context** at FMC compared to the study hospital…

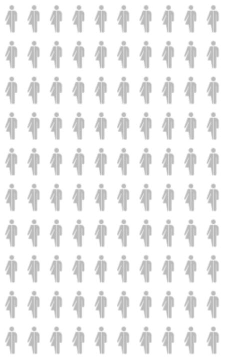

Being optimistic,
at the most how many

out of every 100 hypoglycaemic PoC-BGLs measured would be prevented at FMC?

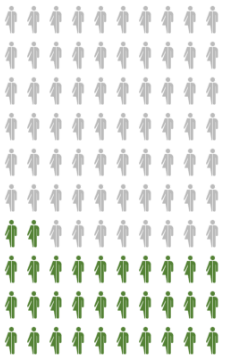

At the study hospital
the intervention prevented

32

of every 100 hypoglycaemic PoC-BGL measurements

Thinking about the **complexity of patients** and the **quality of care** and the **research context** at FMC compared to the study hospital…

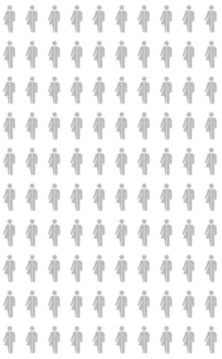

Being pessimistic,
at the least how many

out of every 100 hypoglycaemic PoC-BGLs measured would be prevented at FMC?

Root cause survey (Sinha Gregory 2018)

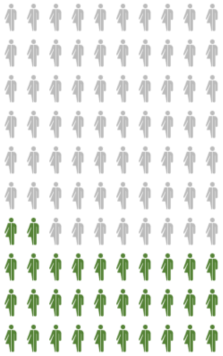

At the study hospital
the intervention prevented

32

of every 100 hypoglycaemic PoC-BGL measurements

Thinking about the **complexity of patients** and the **quality of care** and the **research context** at FMC compared to the study hospital…

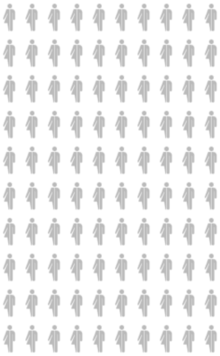

Being realistic,
how many
out of every 100 hypoglycaemic PoC-BGLs measured would be prevented at FMC?

Root cause survey (Sinha Gregory 2018)

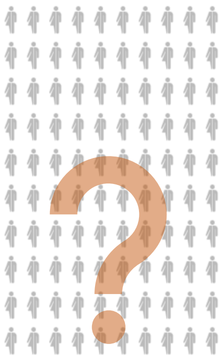

At FMC you estimated the intervention would prevent

? (most realistic estimate)

of every 100 hypoglycaemic PoC-BGL measurements

Turning to the **severe-hypoglycaemia** outcome…

The study did not evaluate the intervention's effect on severe-hypoglycaemia alone.

Root cause survey (Sinha Gregory 2018)

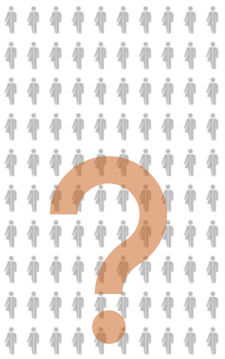

At FMC you estimated the intervention would prevent

? **(most realistic estimate)**

of every 100 hypoglycaemic PoC-BGL measurements

Thinking about **severe hypoglycaemia** compared **hypoglycaemia**…

Do you think the intervention would prevent

more, less or the same

out of every 100 **severely hypoglycaemic** PoC-BGL measurements?

Root cause survey (Sinha Gregory 2018)

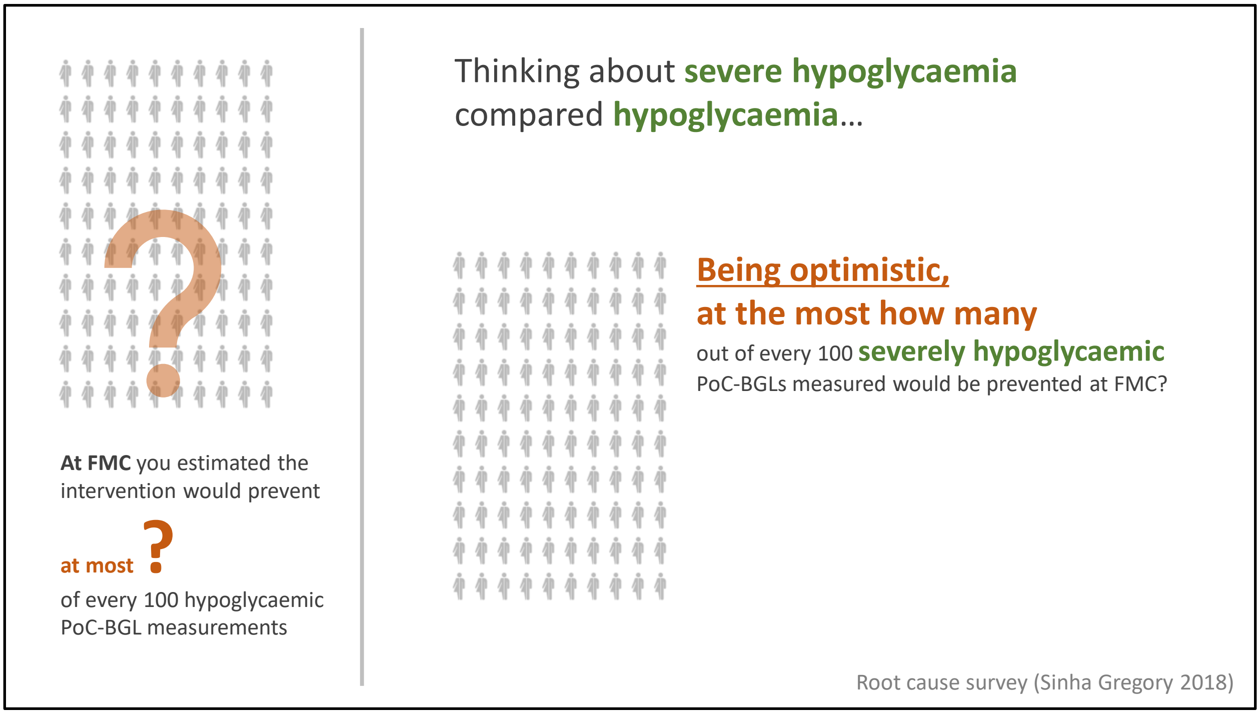

This slide was not needed in session 2 as the experts decided the RR was likely to be the same for hypoglycaemia and severe-hypoglycaemia.

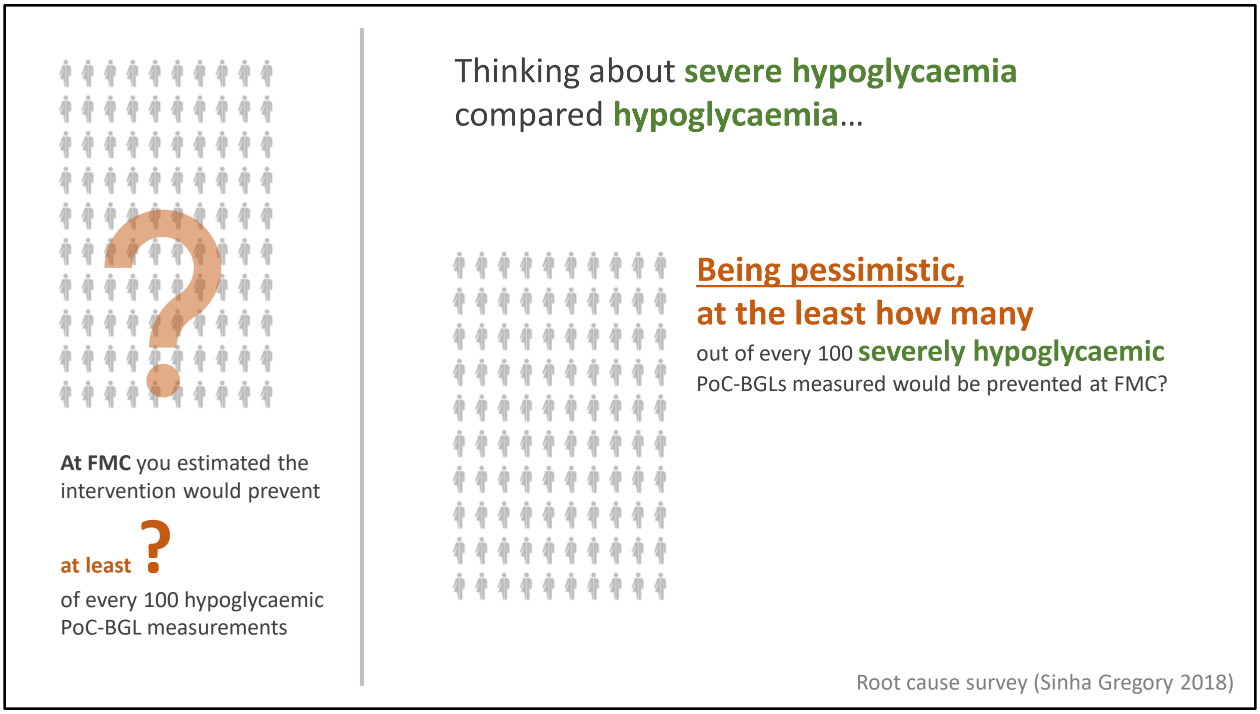

This slide was not needed in session 2 as the experts decided the RR was likely to be the same for hypoglycaemia and severe-hypoglycaemia.

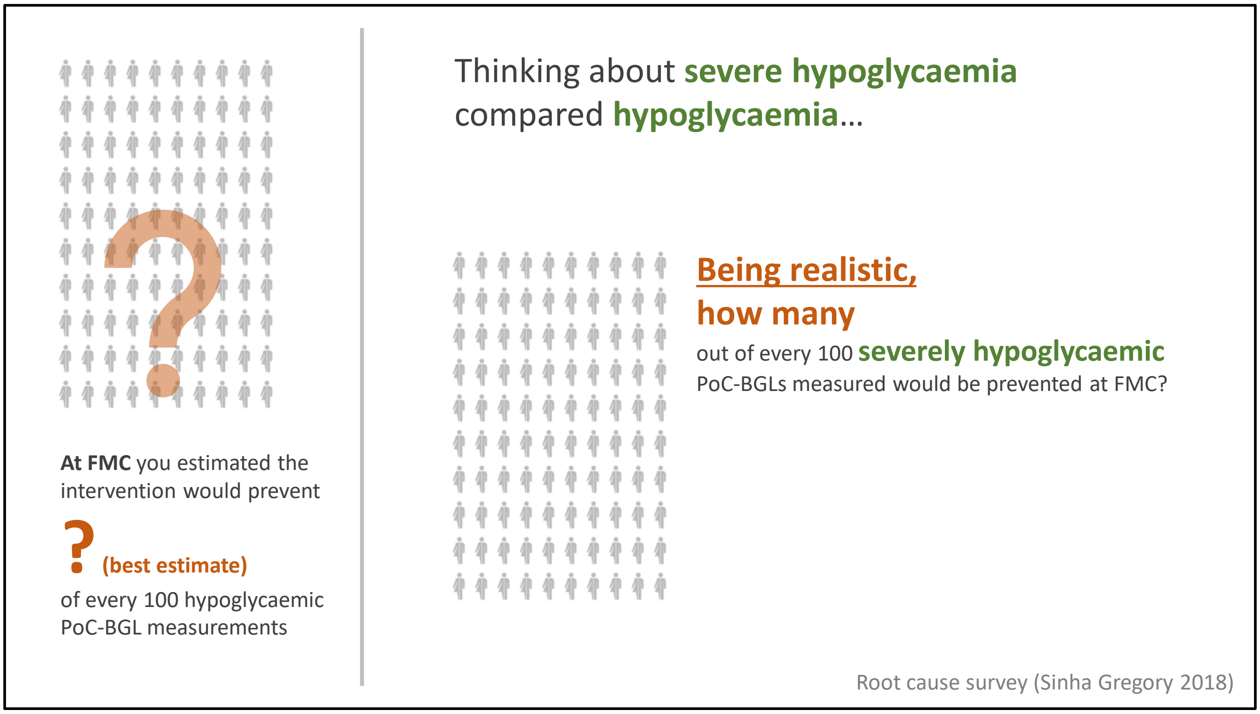

This slide was not needed in session 2 as the experts decided the RR was likely to be the same for hypoglycaemia and severe-hypoglycaemia.
